# A Field‐Deployable RotEx‐LAMP‐LFA Platform for Molecular Triage of HPV‐Driven Oncogenesis

**DOI:** 10.1002/advs.202509468

**Published:** 2025-09-26

**Authors:** Yuan Gao, Qiang Wen, Siyuan Qiao, Linyi Deng, Keyue Li, Zhuyan Shao, Xiaonan Liu, Tao Zhu, Chao Zhang, Da Han, Weihong Tan

**Affiliations:** ^1^ Institute of Molecular Medicine (IMM) Renji Hospital, School of Medicine Shanghai Jiao Tong University Shanghai 200240 China; ^2^ Hangzhou Institute of Medicine (HIM) Zhejiang Cancer Hospital Chinese Academy of Sciences Hangzhou Zhejiang 310022 China; ^3^ School of Forensic Medicine Shanxi Medical University Taiyuan Shanxi 030001 China; ^4^ Molecular Science and Biomedicine Laboratory (MBL) State Key Laboratory of Chemo/Biosensing and Chemometrics College of Chemistry and Chemical Engineering College of Biology Aptamer Engineering Center of Hunan Province Hunan University Changsha Hunan 410082 China

**Keywords:** cervical cancer, diagnostic devices, human papillomavirus, LAMP‐LFA, point‐of‐care testing

## Abstract

Persisting as a major global health disparity, HPV16/18‐associated cervical carcinogenesis continues to disproportionately affect populations in resource‐limited regions with compromised screening infrastructure. While molecular detection of E6/E7 oncogenic transcripts surpasses conventional cytology in clinical specificity, current nucleic acid amplification platforms remain impeded by technical complexity, prolonged turnaround times (>4 h), and substantial per‐test costs (>$50) that hinder scale‐up in low‐income settings. Here, RotEx‐LAMP‐LFA is presented: an innovative point‐of‐care system integrating microfluidic nucleic acid extraction, rapid isothermal amplification (30 min at 63 °C), and combined lateral flow detection in a single disposable cartridge. Validated with 69 clinical specimens (19 histology‐confirmed SCC, 14 HPV+ precancerous lesions, 15 HPV+ infections, and 21 healthy controls), this sample‐to‐answer platform achieved 94.74% sensitivity (18/19; 95% CI: 84.72–100%) for invasive carcinoma detection with 100% (36/36; 95% CI: 90.44‐99.93%) specificity for non‐cancer. In addition, a subset of samples is collected through paired self‐sampling to validate consistency with clinician‐collected specimens, indicating that this innovative platform shows promise for community‐level molecular stratification of persistent and progressive HPV infections. It paves the way for accessible, patient‐centered cervical cancer risk reporting, with the potential to reduce psychological burden on patients.

## Graphical abstract

This study presents RotEx‐LAMP‐LFA, a fully integrated, battery‐powered, low‐cost point‐of‐care device for rapid cervical cancer risk detection based on HPV16/18 E6/E7 mRNA. Clinically validated, the system demonstrated high sensitivity and specificity, and showed full concordance between self‐collected and clinician‐collected samples, offering a promising tool for molecular risk stratification in resource‐limited settings.

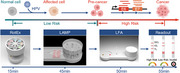

## 1 Introduction

Cervical cancer continues to be a major global health challenge, causing over 0.35 million deaths annually—90% of which occur in low‐ and middle‐income countries (LMICs), where the age‐standardized mortality rate is three times higher than in high‐income regions.^[^ ^1^, ^2^, ^3^ ^]^ In 2020, the World Health Organization (WHO) launched a strategy to eliminate cervical cancer by 2030, with a strong emphasis on large‐scale screening for women within the recommended age range.^[^ ^4^ ^]^ In specific, cervical cancer is largely preventable through a combination of HPV vaccination, systematic screening, and timely treatment of precancerous lesions.^[^ ^5^, ^6^, ^7^ ^]^ It is particularly worth noting that screening plays a critical role in the early detection of cervical cancer, enabling timely intervention and significantly reducing mortality.^[^ ^8^, ^9^ ^]^ However, the implementation of screening programs varies greatly across regions, influenced by differences in socioeconomic conditions, healthcare infrastructure, and education levels.^[^ ^4^, ^10^ ^]^ These disparities highlight the urgent need for low‐cost, accessible screening strategies that can address the specific challenges faced by underserved and resource‐limited populations. Ensuring broader access to effective screening is essential for narrowing health disparities and advancing global efforts to reduce the burden of this largely preventable disease.^[^ ^11^ ^]^

The molecular pathogenesis of HPV‐driven carcinogenesis reveals a key diagnostic challenge: although integration of the HPV16/18 genome into the host genome is known to result in sustained expression of E6/E7 oncoproteins— driving malignant transformation in ≈70% of cases—commonly used screening tools still fail to distinguish transient HPV infections from disease states with progression potential.^[^ ^12^, ^13^ ^]^ For instance, conventional screening methods in LMICs show significant limitations: Pap cytology has a sensitivity of less than 60% for high‐grade lesions in real‐world settings; HPV DNA testing yields a specificity below 60% due to over‐detection of clinically irrelevant infections; and visual inspection methods have inter‐observer agreement rates below 50%.^[^ ^14^, ^15^ ^]^ These limitations result in a pattern of both misdiagnosis and overtreatment, creating a vicious cycle of resource misallocation, diagnostic errors, and delayed interventions—ultimately contributing to preventable deaths that could have been avoided through timely detection and treatment.^[^ ^9^, ^16^ ^]^

The transition from episomal HPV infection to integrated E6/E7 mRNA expression represents a molecular watershed for cervical cancer risk stratification.^[^ ^17^, ^18^ ^]^ Clinical studies have demonstrated that quantitative detection of E6/E7 mRNA yields a positive predictive value of up to 92% for progression to cervical intraepithelial neoplasia grade 3 or higher (CIN3+), compared to only 32% for HPV DNA testing, indicating that E6/E7 mRNA is the most biologically relevant screening target.^[^ ^19^, ^20^ ^]^ However, current mRNA detection platforms, such as RT‐qPCR, TMA, and NASBA, require complex laboratory infrastructure, take over four hours to complete, and incur high per‐test costs, making them difficult to scale in low‐ and middle‐income countries (LMICs).^[^ ^21^, ^22^, ^23^ ^]^ In contrast, research reports on rapid detection methods for HPV DNA have become increasingly diverse and widely available.^[^ ^24^, ^25^, ^26^ ^]^ This technological gap underscores the urgent need for innovative alternatives that meet the WHO target product profile: low‐complexity diagnostic tools suitable for primary care settings with a per‐test cost below $5.^[^ ^27^ ^]^ Moreover, the evaluation of self‐collected vaginal swabs has further expanded the potential for point‐of‐care testing (POCT), offering a more convenient and user‐friendly option for cervical cancer screening.^[^ ^28^ ^]^ Currently, RNA detection in POCT settings primarily relies on methods such as CRISPR,^[^ ^29^, ^30^ ^]^ RPA,^[^ ^31^, ^32^ ^]^ and LAMP.^[^ ^33^, ^34^ ^]^ Among these, RT‐LAMP stands out as particularly suitable for use in primary care environments due to its high specificity and amplification efficiency. However, the inherent instability of RNA and the complexity of sample processing pose significant technical challenges for developing POCT systems targeting HPV E6/E7 mRNA.^[^ ^35^ ^]^ Therefore, there is an urgent need to establish an integrated, low‐power, and visualizable platform capable of stable RNA detection for use in resource‐limited settings.

Here we present RotEx‐LAMP‐LFA, a first‐in‐class rotational microfluidic platform that integrating magnetic bead‐based mRNA capture, isothermal amplification, and semi‐quantitative lateral flow detection into a 45‐min workflow. Designed for community‐level implementation, the system processes self‐collected vaginal swabs through a fully autonomous “sample‐to‐answer” process requiring <10 min of hands‐on time and $4.2 consumable cost (Table S1, Supporting Information). The nucleic acid amplification step can be completed within 30 min, achieving a sensitivity of 6 copies µL^−1^. Among the 69 prospectively enrolled participants, clinical validation showed a sensitivity of 93.33% (95% CI: 84.35–100%) for detecting CIN2+ and a specificity of 100% (95% CI: 90.44–99.93%) for non‐neoplastic cases, compared with the RT‐qPCR reference standard (κ = 0.94, p<0.0001). ROC analysis demonstrated an AUC of 0.94 for stratifying malignant potential. Paired comparison between clinician‐collected and self‐collected samples showed complete concordance. By enabling precise molecular triage at the point of care, this technology supports risk‐adapted resource allocation in line with the WHO's 90‐70‐90 elimination strategy—prioritizing interventions for high‐risk individuals while reducing overtreatment of transient infections.^[^ ^36^ ^]^

## 2 Results and Discussion

### 2.1 RotEx‐LAMP‐LFA: a Point‐of‐Care Platform for Cervical Cancer Risk Stratification

**Figure 1:**
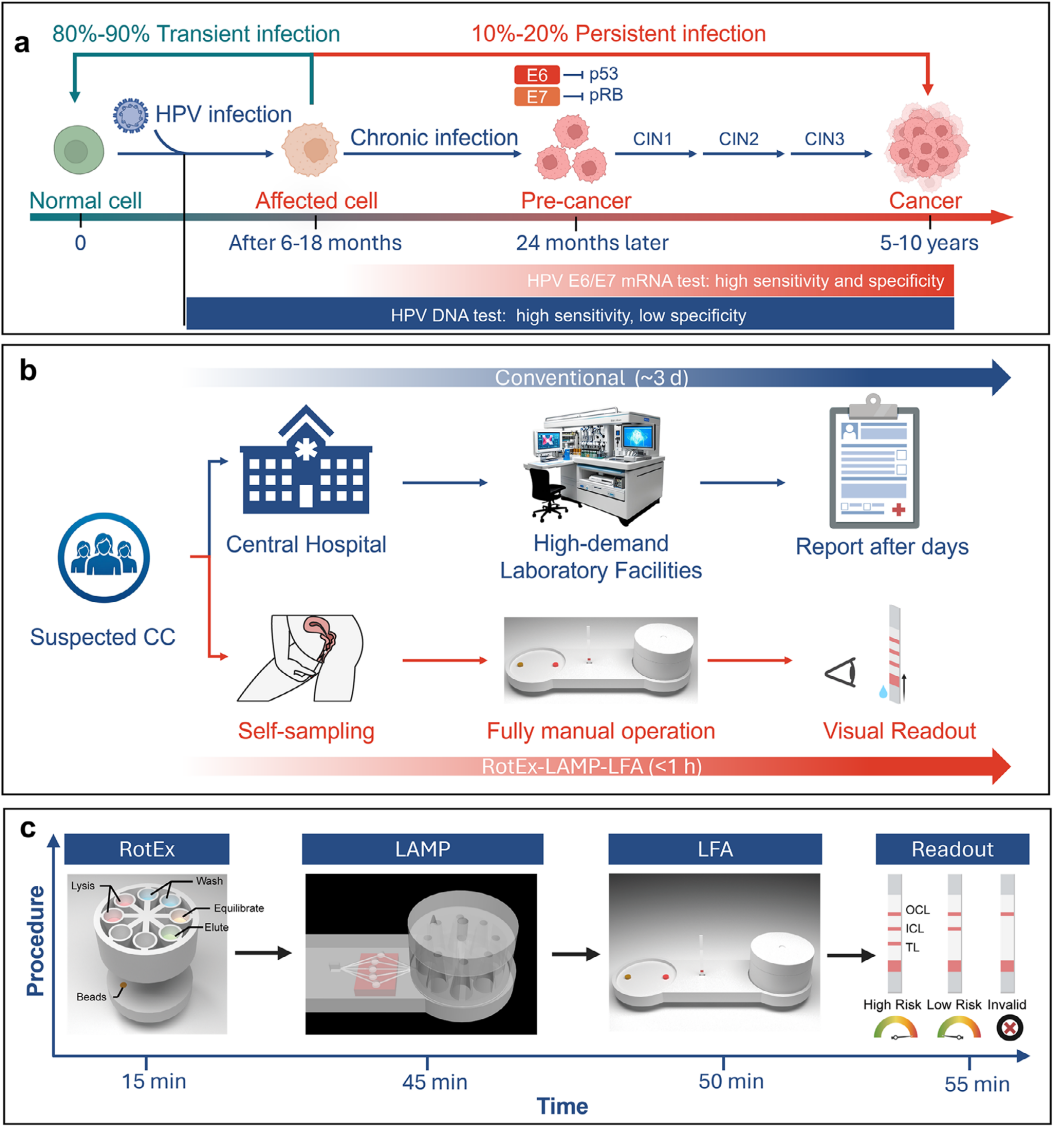
Concept of RotEx‐LAMP‐LFA device for cervical cancer risk assessment. a) HPV‐induced cervical carcinogenesis mediated by sustained E6/E7 oncoprotein expression, highlighting the superior diagnostic specificity of viral mRNA over DNA for detecting neoplastic progression. b) Comparative workflows: Conventional HPV mRNA testing requiring centralized infrastructure (top) versus the self‐contained RotEx‐LAMP‐LFA platform enabling point‐of‐care screening (bottom). c) Device architecture and streamlined operational pipeline integrating self‐sampling, nucleic acid processing, amplification, and visual readout.

Persistent expression of HPV E6 and E7 oncoproteins drives cervical carcinogenesis by degrading tumor suppressors (p53 and pRb) and inducing genomic instability.^[^ ^12^ ^]^ While current clinical practice relies on HPV DNA testing, detection of E6/E7 mRNA provides critical specificity to distinguish transient infections from high‐risk neoplastic progression (**Figure** **1**a).^[^ ^37^ ^]^ To address systemic barriers in global cervical cancer screening—including infrastructure dependency, invasive sampling, and delayed results—we developed the RotEx‐LAMP‐LFA platform, a fully integrated system optimized for resource‐limited settings (Figure 1b).

Conventional HPV mRNA diagnostics necessitate clinician‐collected cervical specimens via speculum‐assisted sampling, a procedure associated with patient discomfort and requiring centralized laboratory infrastructure. These workflows typically involve nucleic acid extraction, reverse transcription‐quantitative PCR (RT‐qPCR), and specialized instrumentation, and delay results by days to weeks. Such delays compromise clinical utility, particularly in LMICs where loss to follow‐up rates exceed 30%.^[^ ^2^, ^38^ ^]^ Our platform circumvents these limitations through four innovations: 1) Patient‐centered self‐sampling: A vaginal self‐collection device optimized for user comfort and effective RNA preservation. 2) Integrated nucleic acid processing: A rotational cartridge preloaded with lyophilized reagents and magnetic beads for mRNA capture from the sample. 3) Isothermal amplification: A battery‐powered heating system that ensures stable reaction conditions for reverse transcription loop‐mediated isothermal amplification (RT‐LAMP). 4) Visual risk stratification: integrated lateral flow strips allow clinician‐independent interpretation, with test line intensity linearly correlated with viral load.

**Figure 2:**
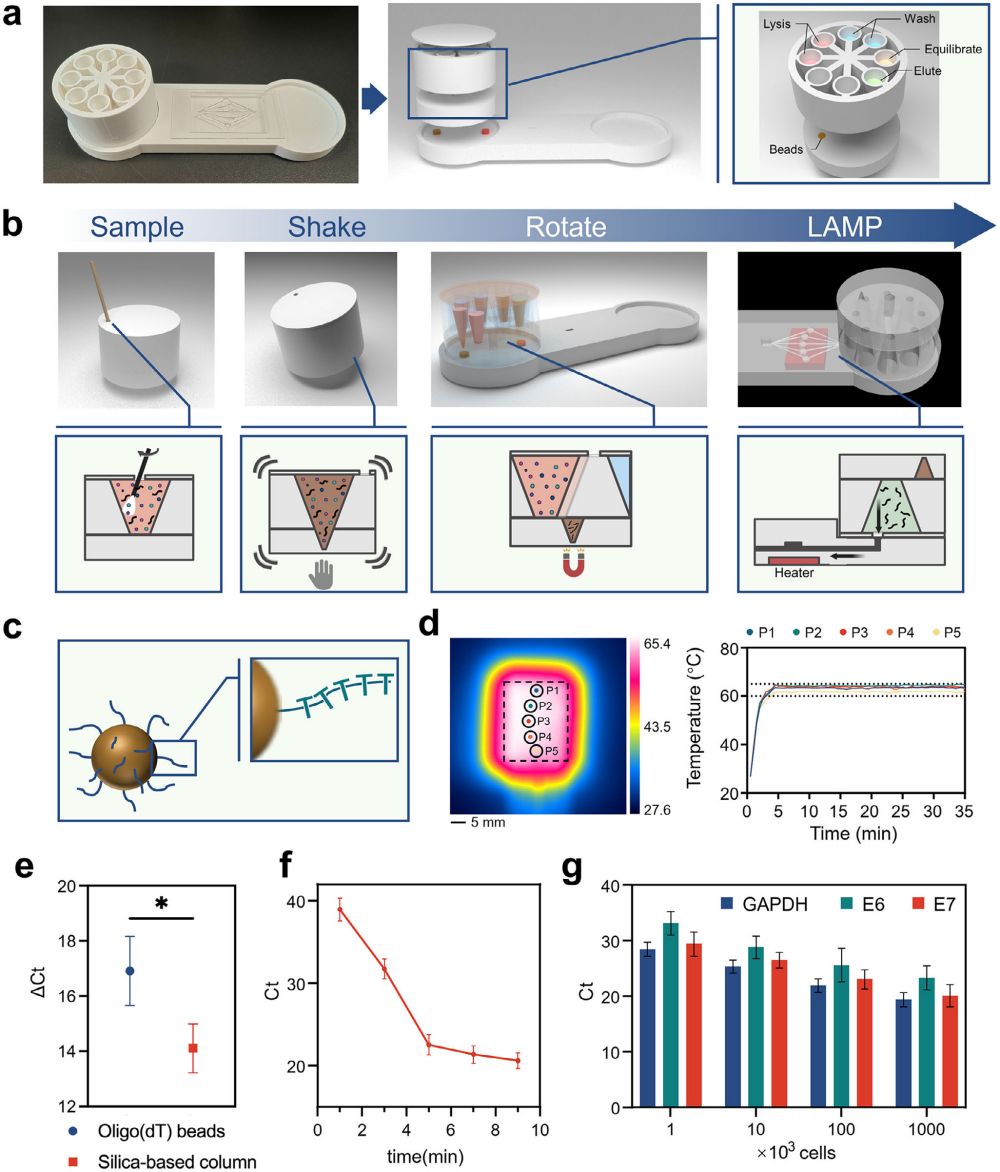
Structural architecture and functional workflow of the RotEx‐LAMP‐LFA system. a) Exploded schematic of the nucleic acid extraction chamber and multifunctional base. b) Schematic and sectional views illustrating the workflow: After sample loading, the process sequentially involves magnetic bead‐mediated RNA capture under manual agitation, reagent alignment, and isothermal nucleic acid amplification. c) Oligo d(T)25‐functionalized paramagnetic bead design for mRNA‐specific binding. d) Thermal characterization of the heating process. The five chambers under the heating unit are used to amplify different target genes (HPV16/18 E6/E7) and the internal control (GAPDH) in parallel. The left panel shows infrared snapshots during the heating process; the dashed line outlines the chip area, and the circles indicate individual heating chambers. The right panel displays real‐time temperature profiles of the five chambers. e) Ct values of the reference gene (GAPDH) with and without reverse transcription across different nucleic acid extraction methods. ΔCt is defined as: Ct(‐RT) – Ct(+RT), the GAPDH Ct value from the sample without reverse transcriptase subtracted from that with reverse transcription. The error bars represent the standard deviations (n = 3). Asterisks denote significance by two‐tailed t‐test (^*^ *P* < 0.05). f) Ct values of GAPDH in samples extracted under different lysis durations. The error bars represent the standard deviations (n = 3). g) Ct values of GAPDH, E6, and E7 in nucleic acids extracted from varying HeLa cell numbers. The error bars represent the standard deviations (n = 3).

As illustrated in **Figure** **2**a, the system consists of a disposable nucleic acid extraction chamber and a reusable multifunctional base, engineered for coordinated use without the need for electrical infrastructure or operator‐specific technical training. The extraction chamber adopts a tri‐layer polypropylene architecture, with a top sealing layer featuring a 5 mm aperture for inserting self‐collected vaginal swabs. The middle layer houses fan‐shaped compartments prefilled with lyophilized reagents—lysis buffer, wash buffer, and elution buffer—while the bottom layer contains paramagnetic beads functionalized with Oligo d(T)25 for sequence‐agnostic poly(A)+ RNA capture. The detailed parameters of the device are provided in the supplementary materials (Figures S2,S3d, Supporting Information).

The workflow begins with inserting the swab into the lysis buffer, followed by manual rotation of the chamber to sequentially align with reagent compartments (Figure 2b). A 30° rotational increment ensures accurate positioning, while 2 Hz agitation for 30 s promotes cell lysis and RNA binding to Oligo d(T)25‐coated magnetic beads (Figure 2c), which selectively capture poly(A)+ mRNA, avoiding genomic DNA contamination (Figure 2e). Integrated N52‐grade neodymium magnets immobilize the beads during buffer exchange, enabling efficient purification without centrifugation or pipetting. To determine the optimal lysis duration, we compared the amount of nucleic acids extracted after 1, 3, 5, 7, and 9 min of lysis (Figure 2f). Based on a balance between extraction efficiency and processing time, the 5‐min lysis yielded the highest efficiency and was selected as the standard condition. In addition, by processing a gradient of cell concentrations, we evaluated the performance of the RotEx system and confirmed its ability to efficiently extract nucleic acids from as few as 1×10^3^ cells (Figure 2g). Furthermore, we investigated the operational robustness of the device, and the results demonstrated that manual shaking can yield stable extraction efficiency within a reasonable range (Figure S2a,b, Supporting Information).

A multifunctional base embeds a self‐regulating PTC heating module (Figure 2d) to ensure uniform temperature across five distinct amplification chambers on the chip, targeting HPV16/18 E6/E7 and the internal control gene GAPDH. By leveraging the intrinsic resistivity change of the PTC material, the module maintains a constant isothermal condition at 63 °C without requiring active electronic feedback. This passive thermal regulation ensures stable performance even under fluctuating power conditions (Figure S3c, Supporting Information).

### 2.2 Systematic Optimization of Integrated RT‐LAMP Assay

**Figure 3:**
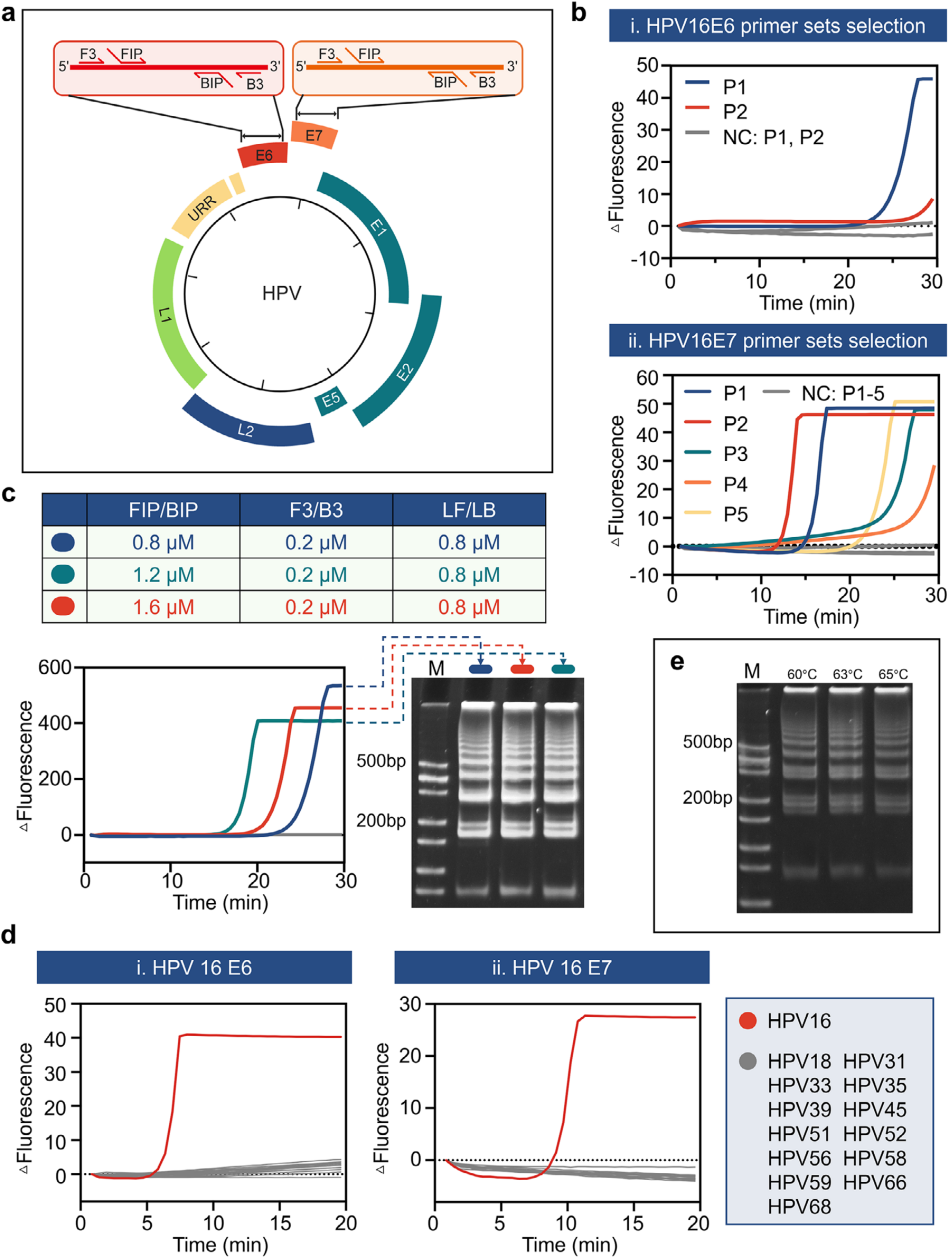
Systematic optimization of RT‐LAMP assays for HPV E6/E7 mRNA detection. a) Genomic localization of HPV16/18 E6/E7 targets and primer binding sites. b) Comparison of fluorescence kinetics curves for different primer pairs targeting HPV16 E6/E7. ΔFluorescence = Fluorescence at each time point‐Initial fluorescence (baseline), represents the change in fluorescence intensity monitored during the amplification reaction. The gray curve represents the no‐template control (NC). c) Evaluation of amplification efficiency under different primer concentration ratios. The table lists the specific concentrations of FIP/BIP, F3/B3, and loop primers used. The gel image on the right shows the visualization of amplification products by agarose gel electrophoresis. “M” indicates the DNA marker (ladder), and the gray band represents the blank control. d) Cross‐reactivity assessment across 14 high‐risk HPV types. e) Temperature tolerance analysis via gel electrophoresis (60 copies µL^−1^). “M” indicates the DNA marker (ladder).

To identify molecular markers associated with tumor progression, we targeted conserved regions of HPV16/18 E6/E7 mRNA (**Figure** **3**a), with GAPDH amplification serving as an internal control for sample adequacy. RT‐LAMP was selected for its operational advantages in resource‐limited settings, combining reverse transcriptase and Bst 3.0 DNA polymerase in a single‐tube reaction to enable RNA‐to‐DNA conversion and exponential amplification at constant temperature.

Primer design prioritized thermodynamic compatibility across global HPV variants, with six candidate sets screened against 238 clinical isolates from the PaVE database (pave.niaid.nih.gov). Primer pairs P1 (HPV16 E6: F3/B3‐FIP/BIP) and P2 (HPV16 E7: F3/B3‐FIP/BIP) exhibited 100% inclusivity, reliably detecting all target variants without cross‐reactivity to 12 non‐target high‐risk HPV types at a concentration of 600 copies µL^−1^ (Figure 3d). Fluorescence kinetics revealed P1 achieved threshold amplification (ΔF > 0.5) in 18.5 ± 2.1 min for HPV16 E6, outperforming suboptimal primers by 42% (Figure 3b). To enhance sensitivity while preserving rapid amplification kinetics, we systematically optimized primer stoichiometry using orthogonal array testing. Final concentrations were set at 0.2 µm for outer primers (F3/B3), 1.6 µm for inner primers (FIP/BIP), and 0.8 µm for loop primers (LF/LB), achieving a balance between minimizing primer‐dimer formation and maximizing amplification efficiency (Figure 3c). Critical to field deployment, the assay demonstrated broad temperature tolerance (60–65 °C) with equivalent amplification efficiency. Gel electrophoresis confirmed consistent laddering patterns across this range (Figure 3e), supporting compatibility with low‐cost heating blocks that lack precise thermal control. Primer screening and concentration optimization for HPV18 E6 and E7 were carried out following a similar workflow (Figures S5–S8, Supporting Information). Contamination risks were mitigated through uracil‐DNA glycosylase (UDG) pretreatment, which degraded 99.98% of carryover amplicons without compromising RNA integrity.^[^ ^39^, ^40^ ^]^

In addition, the combined detection scheme employed differentially labeled primers—FAM for HPV16/18 E6/E7 and DIG for GAPDH—coupled with biotin‐dUTP incorporation. DNA polymerase facilitates elongation using biotin‐labeled dUTP as a substrate (Figure S4, Supporting Information). This design enabled simultaneous lateral flow detection of pathogen and control targets through anti‐FAM/DIG antibody lines, while biotin‐streptavidin interactions immobilized AuNP‐conjugated amplicons for visual readout. Crosstalk between detection channels was eliminated through spatial separation of test lines and stringent wash optimization (Figure S11, Supporting Information). This systematically optimized workflow balances molecular specificity, operational robustness, and field applicability—key requirements for implementing mRNA‐based cervical cancer screening in decentralized healthcare tiers.

### 2.3 Lateral Flow Strip Design for Clinically Actionable Risk Stratification

**Figure 4:**
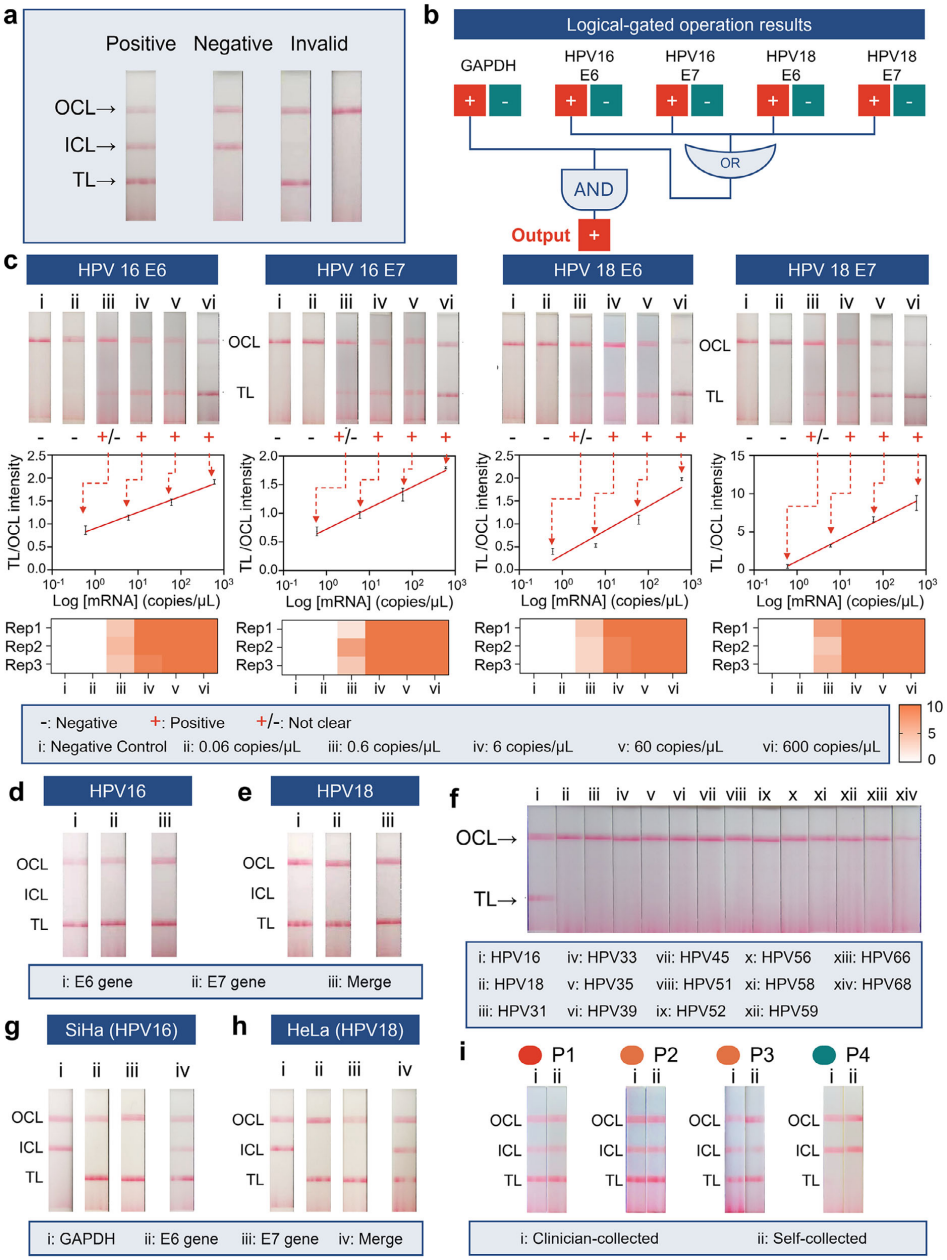
Lateral flow assay optimization and clinical correlation. a) Diagnostic interpretation schema: i) Dual TL/ICL positivity indicates high E6/E7 expression (>6 copies µL^−1^), recommending immediate colposcopy referral. ii) ICL‐only signal suggests low oncogenic activity (<6 copies µL^−1^), supporting routine surveillance. iii) Absence of ICL invalidates the result regardless of TL status, requiring repeat testing. b) Schematic diagram of logic gate operations based on test strip readout. Logical diagram indicating that under the condition where the internal control gene GAPDH is successfully detected, a positive result from any of the HPV E6/E7 targets is interpreted as a high‐risk outcome for cervical cancer. c) Dose‐response relationship between HPV16/18 mRNA concentrations and lateral flow signal intensity (0.06–600 copies µL^−1^). The top panel shows the resulting test strips after reaction; the middle panel presents the linear regression analysis (n = 3), where the x‐axis represents the log‐transformed mRNA concentration and the y‐axis indicates the ratio of TL to OCL band intensities. The bottom panel summarizes the interpretation results by a panel of ten users for three replicate tests. In the heatmap, the color intensity represents the number of individuals who judged the result as positive—darker shades indicate higher perceived positivity. d,e) Diagnostic results for (d) HPV16 and (e) HPV18 E6/7 RNA pseudoviruses, illustrating the optimization of the lateral flow assay. f) Specificity validation against 14 non‐target hrHPVs (60 copies µL^−1^). g‐h) Cell line validation using SiHa (HPV16+) and HeLa (HPV18+) lysates (100 cells/reaction). i) Strip readout from paired clinician‐collected and matched self‐collected samples from the same patients.

The lateral flow strip was engineered to convert molecular amplification signals into visually interpretable clinical decisions through a combined capture architecture (**Figure** **4**a). Biotinylated LAMP amplicons complex with streptavidin‐conjugated gold nanoparticles (SA‐AuNPs) via high‐affinity interaction, while FAM‐labeled HPV targets and DIG‐tagged GAPDH amplicons are captured by immobilized anti‐FAM (TL) and anti‐DIG (ICL) antibodies, respectively. Excess SA‐AuNPs are sequestered at the outer control line (OCL) through biotin‐SA conjugation, creating a three‐line validation system that confirms both assay functionality and specimen adequacy (Figure S9, Supporting Information). Representative visual results of the test strips are shown in Figure 4a. These results can be interpreted using logic gate operations based on multiple gene targets (Figure 4b).

Analytical sensitivity was established using serial dilutions of HPV16/18 E6/E7 mRNA standards (0.06–600 copies µL^−1^) (Figure 4c). Dose‐response analysis revealed linear correlations between TL intensity (quantified via ImageJ) and viral load (R^2^ = 0.96 and 0.96 for HPV16 E6/E7, R^2^ = 0.90 and 0.97 for HPV18 E6/E7), enabling semi‐quantitative risk stratification. Additionally, a panel of ten users was invited to independently interpret the results of three replicate tests for each target at each concentration. A clear linear correlation was maintained across a 5‐fold serial dilution range (1.2‐750 copies µL^−1^), further validating the assay's quantitative reliability (Figure S10, Supporting Information). Positive interpretations remained consistent for concentrations ranging from 6 to 600 copies µL^−1^, while the results at 0.6 copies µL^−1^ were more ambiguous. Furthermore, to statistically determine the assay's sensitivity, we performed multiple replicates at 6 copies µL^−1^ for all four targets and established a limit of detection (LoD) of 6 copies µL^−1^ (≥95% positive detection, 40 replicates). Specifically, 39 out of 40 replicates were positive for HPV16E6, 40/40 for HPV16E7, 38/40 for HPV18E6, and 39/40 for HPV18E7 (Table S2, Supporting Information). This level of sensitivity is comparable to that of the FDA‐approved Aptima HPV assay, but as a POCT platform, this platform eliminates the need for bulky instrumentation.

Specificity was validated against synthetic plasmids encoding E6/E7 genes from 14 non‐target hrHPVs (60 copies µL^−1^), including phylogenetically related types HPV31/33/35.^[^ ^41^ ^]^ No cross‐reactivity was observed (Figure 4f; Figure S11, Supporting Information), confirming the primer set's selectivity for HPV16/18.

Prior to testing cellular samples, the system was validated using HPV E6/E7 mRNA pseudovirus following plasmid construction verification (Figure 4d–e). Standard templates were amplified with E6 and E7 primers, and the results were visualized on both individual and combined test strips. Preclinical validation using HPV+ cell lines further demonstrated clinical relevance: SiHa (HPV16+) and HeLa (HPV18+) lysates generated robust TL signals at 100 cells/reaction, while GAPDH amplification confirmed specimen integrity across all tests (Figure 4g,h). The above validation was performed using known samples to assess the accuracy and reliability of the RT‐LAMP and LFA components of the system.

### 2.4 Clinical Validation of the RotEx‐LAMP‐LFA System

To assess the reliability of the RotEx‐LAMP‐LFA system for cervical cancer screening, we validated its predictive accuracy using clinical samples, including self‐collected vaginal secretions and healthcare professional‐collected samples under standard protocols as controls.

**Figure 5:**
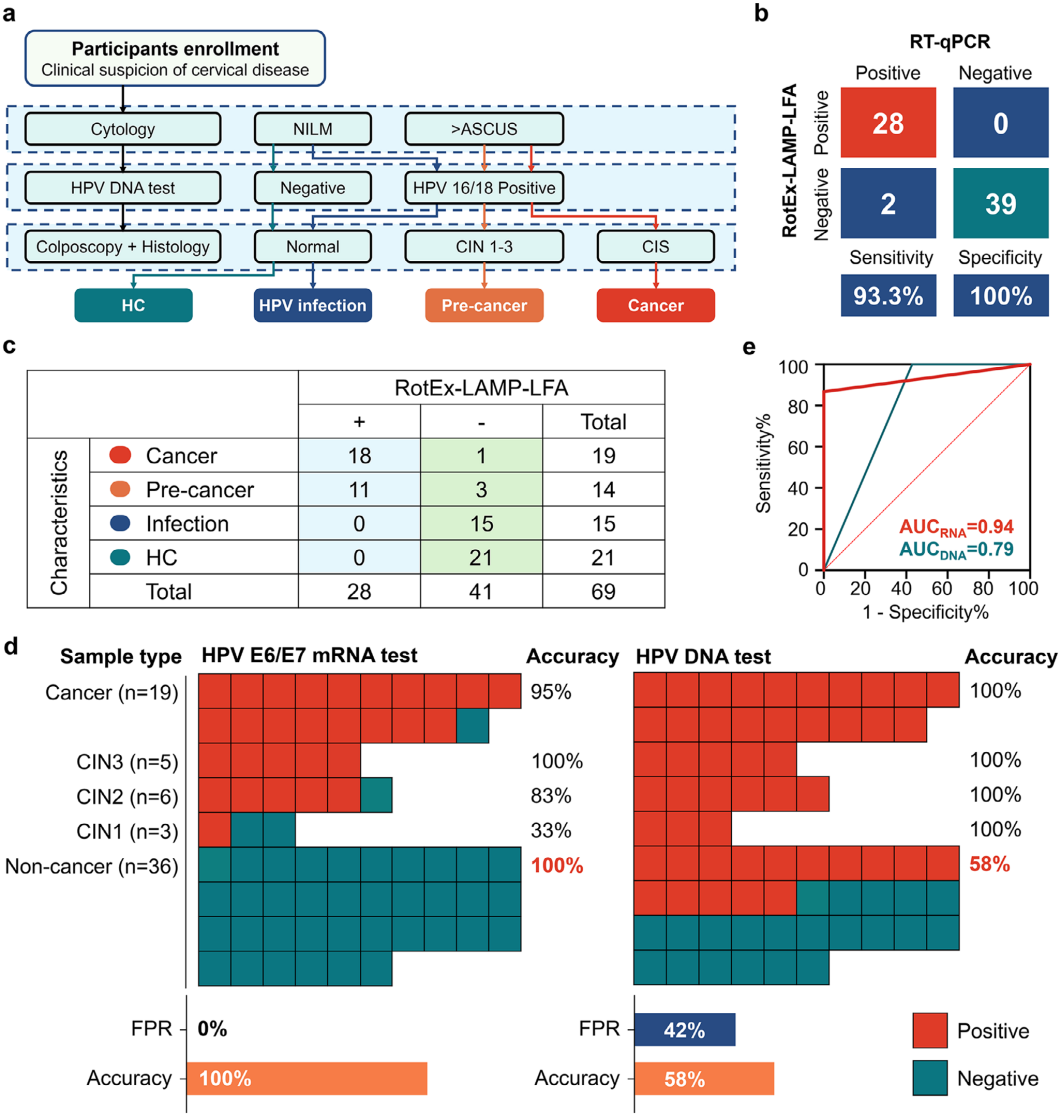
Summary of diagnostic results from clinical samples. a) Flowchart of patient grouping. Based on cytology, HPV DNA testing, colposcopy, and histology results, the cohort was divided into healthy controls (HC), HPV infection, precancerous lesions (Pre‐cancer), and Cancer. b) Sensitivity and specificity of the RotEx‐LAMP‐LFA system were evaluated by comparing its results with RT‐qPCR, using a confusion matrix. c) Statistical analysis of clinical sample testing results. d) Comparison of clinical sample results obtained from RotEx‐LAMP‐LFA and HPV DNA testing. CIN: cervical intraepithelial neoplasia, CIN grading reflects the extent of epithelial abnormality, with CIN2 and above (CIN2+) indicating high‐grade lesions that warrant clinical management due to their strong correlation with cervical cancer progression; FPR: false positive rate. e) ROC analysis comparing the ability of RotEx‐LAMP‐LFA and HPV DNA testing to distinguish CIN2+ risk.

The study cohort comprised patients undergoing standard cervical cancer diagnostic evaluations during hospital visits, including cytological exams, HPV DNA testing, and/or colposcopy. Histological examinations of cervical biopsies were performed if colposcopy revealed abnormalities. Based on diagnostic outcomes, participants were categorized into four groups: 1) the tumor group, consisting of HPV DNA 16/18‐positive patients with cytological findings of atypical squamous cells of undetermined significance (ASCUS) or higher and histologically confirmed cervical squamous cell carcinoma; 2) the precancerous lesion group, including patients with histologically confirmed cervical intraepithelial neoplasia (CIN) grades 1–3; 3) the HPV infection group, comprising HPV DNA 16/18‐positive patients with normal cytology (no intraepithelial lesion or malignancy, NILM) and no biopsy abnormalities; and 4) the healthy control (HC) group, consisting of patients with negative or normal results across all diagnostic tests. The final study cohort included 69 individuals: 19 cervical cancer patients, 14 cases of CIN, 15 non‐cancer individuals positive for HPV16/18 DNA, and 21 HPV16/18 DNA‐negative controls (Table S6, Supporting Information). In addition, using RT‐qPCR as the reference standard to detect HPV16 and HPV18 E6/E7 mRNA, the RotEx system demonstrated a sensitivity of 93.3% (28/30 positive samples correctly identified) and a specificity of 100.0% (39/39 negative samples correctly identified) (**Figure** **5**b), showing near‐complete agreement between the two methods (97.1% (67/69) Accuracy). The slight discrepancy in sensitivity may stem from the inherently lower sensitivity of LAMP compared to RT‐qPCR.

The RotEx‐LAMP system accurately reflected cervical lesion severity, achieving a sensitivity of 95% (18/19) for cervical cancer detection and a detection rate of 79% (11/14) for precancerous lesions. Only one HPV‐infected case without malignant transformation was identified, while the true negative rate for the HC group was 100%. Receiver operating characteristic (ROC) curve analysis showed that the area under the curve (AUC) for CIN2+ risk prediction based on HPV E6/E7 mRNA detection was 0.94 (Figure 5e), compared to 0.74 for HPV DNA testing. CIN grading reflects the extent of epithelial abnormality, with CIN2 and above (CIN2+) indicating high‐grade lesions that warrant clinical management due to their strong correlation with cervical cancer progression. These results indicate that the mRNA‐based system enables rapid and reliable assessment of cervical cancer risk and is less affected by transient HPV infections than DNA‐based methods. In contrast, 15 patients who tested positive by HPV DNA testing might have been subjected to unnecessary follow‐up procedures. The RotEx system helps reduce overdiagnosis, improving the accuracy of non‐cancer detection from 58% (21/36) to 100% (36/36), and reducing the false‐positive rate from 42% to 0% (Figure 5d). Moreover, when analyzing precancerous lesions in more detail, the mRNA‐based positive detection rate showed a trend correlating with the severity of CIN grading—from 33% in CIN1, to 83% in CIN2, and 100% in CIN3—whereas DNA‐based testing did not exhibit this pattern.

To evaluate the performance of the system in self‐sampling scenarios, we collected four pairs of clinician‐collected and self‐collected samples, and tested them using the integrated RotEx‐LAMP platform (Figure 4i). The cohort included one cervical cancer patient (P1), two patients with precancerous lesions (P2 and P3), and one HC (P4). The results demonstrated complete concordance between the two sampling methods and accurately reflected the varying levels of cervical cancer risk, highlighting the potential of this system as a POCT tool.

## 3 Conclusion and Discussion

Cervical cancer remains a preventable disease, yet its global burden persists due to systemic gaps in accessible, accurate, and timely screening—particularly in low‐resource settings. To address these challenges, we developed the RotEx‐LAMP‐LFA platform—an integrated diagnostic system that combines patient‐centered self‐sampling, battery‐powered nucleic acid processing, and rapid visual readout. This platform integrates oligo d(T)‐functionalized paramagnetic bead‐based RNA capture, RT‐LAMP amplification, and integrated lateral flow readout into a streamlined workflow. Key innovations include a self‐regulating PTC heating module for ambient‐temperature amplification and a co‐linear lateral flow strip that encodes molecular complexity into a simple visual triage logic. The cartridge features a closed, user‐friendly design requiring only lysis, mixing, incubation, and strip insertion, making it operable by non‐experts and suitable for self‐sampling. Notably, simultaneous detection of four HPV E6/E7 targets is achieved without added procedural complexity, enhancing interpretability and diagnostic robustness. The complete concordance observed in paired self‐collected samples underscores the platform's potential to reduce psychological stress and financial burdens associated with clinic‐based testing, thereby improving screening accessibility and compliance in low‐resource or at‐home settings.

By targeting HPV16/18 E6/E7 mRNA—the molecular drivers of cervical carcinogenesis—the platform achieves clinic‐grade accuracy (95.6% sensitivity, 100% specificity) while eliminating dependencies on centralized laboratories and specialized training. The system's closed, rotationally controlled workflow enables users to complete screening in under one hour, at a total device cost of $10–15 (Table S1, Supporting Information). Current HPV detection methods remain relatively costly, which limits their scalability and accessibility in resource‐limited settings. We anticipate that future large‐scale manufacturing could further reduce the per‐test cost, facilitating wider adoption in low‐resource clinical contexts. Clinical validation in a prospective cohort (n = 69) demonstrated robust discrimination of high‐grade lesions (AUC = 0.94) and 100% specificity in differentiating oncogenic progression from transient HPV infections, addressing a critical limitation of current DNA‐based screening paradigms.

Key innovations include Oligo d(T)‐functionalized paramagnetic beads for direct mRNA capture, a self‐regulating PTC heating module for ambient‐temperature amplification, and an integrated lateral flow strip encoding molecular complexity into visual triage logic. The complete concordance observed in paired self‐collected samples highlights the platform's potential to significantly reduce patients’ psychological stress and financial burden associated with clinical visits, thereby improving compliance.

To support the WHO's “90‐70‐90” cervical cancer elimination strategy, this system could play a critical role in regions where screening accessibility is hindered by limited infrastructure and cultural barriers. Our initial objective was to enable diagnostic testing by medical teams operating in resource‐limited settings, such as remote areas lacking access to large‐scale equipment or even a stable power supply. However, field testing in these settings is still necessary to validate the operational reliability of the platform under diverse environmental conditions. While the current workflow is designed to be operable by personnel with basic training, we aim to further streamline the procedure in future iterations, with the long‐term goal of expanding its applicability to home‐based self‐testing.

Future efforts will focus on collaborating with national health systems to implement large‐scale screening programs, integrating telemedicine platforms to assist with result interpretation, and expanding detection capabilities to cover additional hrHPV genotypes. This study presents a clear blueprint for the next generation of decentralized diagnostic technologies, demonstrating that high technical sophistication can be compatible with real‐world usability—a key step toward achieving global health equity in oncology.

## 4 Experimental Section

### Primer Design and Oligonucleotide Preparation

LAMP primers targeting HPV16/18 E6/E7 transcripts were designed using PrimerExplorer V5 (Eiken Chemical Co., Japan). GAPDH primers were adapted from a validated source.^[^ ^42^ ^]^ All oligonucleotides (Tables S3,S4, Supporting Information) were synthesized by Sangon Biotech (Shanghai, China), dissolved in nuclease‐free TE buffer (pH 8.0) at 10 µm, and stored at –20 °C until use.

### RT‐LAMP Amplification

RT‐LAMP was carried out using the WarmStart^®^ LAMP Kit (New England Biolabs, Cat# E1700S). Reactions included 0.2 µm outer primers (F3/B3), 0.8 µm inner primers (FIP/BIP), and 20 µm Biotin‐11‐dUTP (Thermo Fisher) for lateral flow compatibility. Amplification was performed at 63 °C for 30 min on a LightCycler 480 II (Roche Diagnostics) to monitor kinetics. Products were stored at 4 °C for up to 24 h before further analysis.

### RT‐qPCR Quantification

Total RNA was reverse transcribed into cDNA using a commercial RT kit (Takara, Cat# RR037Q), followed by qPCR using Takara qPCR Mix (Cat# RR820L). Amplification was monitored on a LightCycler 480 II, and Ct values were recorded.

### Electrophoretic Validation

RT‐LAMP products (5 µL) were mixed with DNA loading buffer (1 µL, 6×) and electrophoresed on 10% native PAGE at 110 V for 45 min in 1× TBE buffer. Gels were stained with GelRed™ (3×, Sangon Biotech) and visualized using a Gel Doc EZ system (Bio‐Rad) with Image Lab v6.1.

### Specificity Testing

Assay specificity was tested using synthetic plasmids encoding E6/E7 genes from 14 high‐risk HPV genotypes (data from PaVE, NIH). Plasmids (Sangon Biotech, Table S4, Supporting Information) were diluted to 40 ng µL^−1^ in TE buffer and kept at –20 °C.

### Lateral Flow Detection

For visual detection, 2 µL of biotinylated product was applied to a lateral flow strip (Ustar, China) with streptavidin and capture probes. Strips were incubated in 100 µL of buffer for 3 min. The upper control line confirmed LFA validity, the internal line confirmed sample processing, and the test line indicated target detection.

### Pseudovirus and Cellular Models

HPV16/18 E6/E7 pseudoviruses (NIM‐RM 5231, 5232) were obtained from the National Institute of Metrology (Beijing, China) and stored at –80 °C. SiHa and HeLa cell lines (CTCC‐001‐0006 and CTCC‐001‐0629) were supplied by MeisenCTCC and cultured under standard conditions.

### RNA Extraction Methods

mRNA was extracted using Oligo d(T)‐coated magnetic beads (NEB, Cat# S1550S) following the manufacturer's instructions. Samples were lysed, incubated with beads for hybridization, washed sequentially, and eluted in elution buffer after equilibration. For DNA contamination comparison, RNA Easy Fast Tissue/Cell Kits (TIANGEN, Cat# DP451) were also tested per manufacturer protocols.

### Clinical Specimen Processing

This study was approved by the Ethics Committee of Shanghai Renji Hospital (approval no. KY2025‐004‐A), and written informed consent was obtained from all participants prior to the collection of clinician‐collected and guided self‐collected vaginal swab samples. A total of 69 clinician‐collected swabs were obtained during routine examinations and placed into tubes containing preservation solution. Additionally, 4 participants were guided to perform self‐collection using HPV kits (HCYTECH, Shenzhen, China; Figure S1a, Supporting Information). All specimens were processed within 2 h and stored at –80 °C until analysis.

The sample detection process was as follows: The collected swab was first eluted in the first sector of the RotEx extraction chamber. The chamber was then manually rotated to allow the magnetic beads to sequentially contact different reagents, followed by hand‐shaking to complete the sample extraction. The distribution of reagents in each sector is shown in Figure S3d (Supporting Information). During this process, the extraction chamber was placed in a designated position on the device base, where magnets embedded in the base facilitated the precipitation of magnetic beads. This enabled a stepwise protocol including lysis, washing, equilibration, and final elution. After extraction, the chamber was inverted and aligned with the amplification chip for the subsequent RT‐LAMP reaction. Once aligned, the isothermal heating module was activated to initiate amplification. Upon completion, a lateral flow strip was dipped into the reaction product solution for visual readout.

For mRNA detection by RT‐qPCR, a portion of the swab preservation solution after vortex mixing was taken. Total mRNA was extracted following the procedure described above, then reverse‐transcribed into cDNA, followed by fluorescence signal monitoring using qPCR.

### Statistical Analysis

All clinical data were analyzed using GraphPad Prism version 9.5.0. Data are expressed as mean ± SD based on at least three independent biological replicates unless otherwise stated. Comparisons between two groups were performed using an unpaired, two‐tailed Student's t‐test. Sample sizes (n) refer to independent biological replicates and are reported for each analysis in the figure legends or relevant Methods subsections. Diagnostic performance was assessed by plotting receiver operating characteristic (ROC) curves, with calculations of AUC, accuracy, and sensitivity. Confidence intervals for proportion estimates in chi‐square tests were determined using the Wald and Wilson methods. A *p*‐value of less than 0.05 was considered statistically significant.

## Conflict of Interest

The authors declare no conflict of interest.

## Supporting information



Supporting Information

## Data Availability

The data that support the findings of this study are available from the corresponding author upon reasonable request.

## References

[1] F. Bray , M. Laversanne , H. Sung , J. Ferlay , R. L. Siegel , I. Soerjomataram , A. Jemal , CA A Cancer J Clinicians 2024, 74, 229. 10.3322/caac.21834 38572751

[2] L. Rahangdale , C. Mungo , S. O'Connor , C. J. Chibwesha , N. T. Brewer , BMJ 2022, 379, 070115. 10.1136/bmj-2022-070115 36521855

[3] A. A. Francoeur , B. J. Monk , K. S. Tewari , Nat. Rev. Clin. Oncol. 2025, 22, 182. 39753753 10.1038/s41571-024-00977-w

[4] T. Malagón , E. L. Franco , R. Tejada , S. Vaccarella , Nat. Rev. Clin. Oncol. 2024, 21, 522. 38760499 10.1038/s41571-024-00904-z

[5] M. Falcaro , A. Castañón , B. Ndlela , P. Sasieni , The Lancet Regional Health – Europe 2025, 49, 101157. 39759575 10.1016/j.lanepe.2024.101157 PMC11697118

[6] F. Wei , D. Georges , I. Man , I. Baussano , G. M. Clifford , Lancet 2024, 404, 435. 39097395 10.1016/S0140-6736(24)01097-3

[7] R. B. Perkins , N. Wentzensen , R. S. Guido , M. Schiffman , C. C. S.: A. Review, JAMA 2023, 330, 547. 10.1001/jama.2023.13174 37552298

[8] R. A. Smith , K. S. Andrews , D. Brooks , S. A. Fedewa , D. Manassaram‐Baptiste , D. Saslow , R. C. Wender , CA A Cancer J Clinicians 2019, 69, 184. 10.3322/caac.21557 30875085

[9] P. E. Castle , M. H. Einstein , V. V. Sahasrabuddhe , CA A Cancer J Clinicians 2021, 71, 505. 10.3322/caac.21696 PMC10054840 34499351

[10] G. F. Sawaya , K. Smith‐McCune , M. Kuppermann , JAMA, J. Am. Med. Assoc. 2018, 321, 2019. 10.1001/jama.2019.4595 PMC6656358 31135834

[11] G. Mohan , S. Chattopadhyay , JAMA Oncol 2020, 6, 1434. 32556187 10.1001/jamaoncol.2020.1460 PMC7857975

[12] A. A. McBride , Nat. Rev. Microbiol. 2022, 20, 95. 34522050 10.1038/s41579-021-00617-5

[13] M. Florence , D. R. Flum , G. J. Jurkovich , P. Lin , S. R. Steele , R. G. Symons , R. Thirlby , Ann. Surg. 2008, 248, 557. 18936568 10.1097/SLA.0b013e318187aeca

[14] L. E. Markowitz , E. R. Unger , N. Engl. J. Med. 2023, 388, 1790. 37163625 10.1056/NEJMcp2108502 PMC11567082

[15] M. Gultekin , P. T. Ramirez , N. Broutet , R. Hutubessy , Int J Gynecol Cancer 2020, 30, 426. 32122950 10.1136/ijgc-2020-001285

[16] K. S. Kechagias , I. Kalliala , S. J. Bowden , A. Athanasiou , M. Paraskevaidi , E. Paraskevaidis , J. Dillner , P. Nieminen , B. Strander , P. Sasieni , A. A. Veroniki , M. Kyrgiou , BMJ 2022, 378, 070135. 10.1136/bmj-2022-070135 PMC9347010 35922074

[17] M. A. Molina , R. D. M. Steenbergen , A. Pumpe , A. N. Kenyon , W. J. G. Melchers , Trends Mol. Med. 2024, 30, 890. 38853085 10.1016/j.molmed.2024.05.009

[18] M. Pett , N. Coleman , J. Pathol. 2007, 212, 356. 17573670 10.1002/path.2192

[19] M. Arbyn , M. Simon , S. De Sanjosé , M. A. Clarke , M. Poljak , R. Rezhake , J. Berkhof , V. Nyaga , M. Gultekin , K. Canfell , N. Wentzensen , Lancet Oncol. 2022, 23, 950. 35709810 10.1016/S1470-2045(22)00294-7

[20] P. K. Pretsch , Lancet Public Health 2023, 8, 411.

[21] M. Arbyn , M. Simon , E. Peeters , L. Xu , C. J. L. M. Meijer , J. Berkhof , K. Cuschieri , J. Bonde , A. Ostrbenk Vanlencak , F.‐H. Zhao , R. Rezhake , M. Gultekin , J. Dillner , S. De Sanjosé , K. Canfell , P. Hillemanns , M. Almonte , N. Wentzensen , M. Poljak , Clin. Microbiol. Infect. 2021, 27, 1083. 33975008 10.1016/j.cmi.2021.04.031

[22] M. Arbyn , P. J. F. Snijders , C. J. L. M. Meijer , J. Berkhof , K. Cuschieri , B. J. Kocjan , M. Poljak , Clin. Microbiol. Infect. 2015, 21, 817. 25936581 10.1016/j.cmi.2015.04.015

[23] P. J. Toliman , J. M. Kaldor , S. G. Badman , S. Phillips , G. Tan , J. M. L. Brotherton , M. Saville , A. J. Vallely , S. N. Tabrizi , Clin. Microbiol. Infect. 2019, 25, 496. 29906593 10.1016/j.cmi.2018.05.025

[24] J. Zhang , Z. Dong , L. Xu , X. Han , Z. Sheng , W. Chen , J. Zheng , D. Lai , F. Shen , Adv. Sci. 2024, 11, 2406367. 10.1002/advs.202406367 PMC11578293 39320328

[25] H. Bai , Y. Liu , L. Gao , T. Wang , X. Zhang , J. Hu , L. Ding , Y. Zhang , Q. Wang , L. Wang , J. Li , Z. Zhang , Y. Wang , C. Shen , B. Ying , X. Niu , W. Hu , Biosens. Bioelectron. 2024, 248, 115968. 38150799 10.1016/j.bios.2023.115968

[26] K. A. Kundrod , M. Barra , A. Wilkinson , C. A. Smith , M. E. Natoli , M. M. Chang , J. B. Coole , A. Santhanaraj , C. Lorenzoni , C. Mavume , H. Atif , J. R. Montealegre , M. E. Scheurer , P. E. Castle , K. M. Schmeler , R. R. Richards‐Kortum , Sci. Transl. Med. 2023, 15, abn4768. 10.1126/scitranslmed.abn4768 PMC10566637 37343083

[27] World Health Organization , Target Product Profiles for Human Papillomavirus Screening Tests to Detect Cervical Pre‐cancer and Cancer,World Health Organization, Geneva 2024.

[28] J. Lei , K. Cuschieri , H. Patel , A. Lawrence , K. Deats , P. Sasieni , A. W. W. Lim , PLoS Med. 2024, 21, 1004494. 10.1371/journal.pmed.1004494 PMC11637256 39666756

[29] T. Hall , S. Gulati , R. Sang , Z. Jia , F. Mckinnirey , G. Vesey , E. Goldys , F. Deng , TrAC Trends in Analytical Chemistry 2025, 118275.

[30] J. Zhong , J. Li , S. Chen , Y. Xu , X. Mao , M. Xu , S. Luo , Y. Yang , J. Zhou , J. Yuan , L. Su , G. Wang , X. Zhang , X. Li , J. Appl. Microbiol. 2025, 136, lxaf070. 40097290 10.1093/jambio/lxaf070

[31] P. Bhardwaj , P. Dhangur , A. Kalichamy , R. Singh , J. Med. Virol. 2025, 97, 70219. 10.1002/jmv.70219 39949262

[32] Y. Guo , T. Ge , Q. Wang , T. Liu , Z. Li , Insect Science 2025, 1744. 10.1111/1744-7917.70024 40098415

[33] S. D. Villota , E. Veloz‐Villavicencio , S. Garcia‐Iturralde , J. V. Arévalo , S. Lu , K. Jaenes , Y. Guo , S. Cicek , K. Colwill , A.‐C. Gingras , R. Bremner , P. Ponce , K. Pardee , V. E. Cevallos , PLoS One 2025, 20, 0321794. 10.1371/journal.pone.0321794 PMC12002511 40238804

[34] C. Zhang , T. Zheng , H. Wang , W. Chen , X. Huang , J. Liang , L. Qiu , D. Han , W. Tan , Anal. Chem. 2021, 93, 3325. 33570399 10.1021/acs.analchem.0c05059

[35] O. Fashedemi , O. C. Ozoemena , S. Peteni , A. B. Haruna , L. J. Shai , A. Chen , F. Rawson , M. E. Cruickshank , D. Grant , O. Ola , K. I. Ozoemena , Anal. Methods 2025, 17, 1428. 39775553 10.1039/d4ay01921k PMC11706323

[36] H. Sung , J. Ferlay , R. L. Siegel , M. Laversanne , I. Soerjomataram , A. Jemal , F. Bray , CA Cancer J Clin 2021, 71, 209. 33538338 10.3322/caac.21660

[37] P. A. Cohen , A. Jhingran , A. Oaknin , L. Denny , C. cancer , Lancet 2019, 393, 169. 30638582 10.1016/S0140-6736(18)32470-X

[38] M. Xu , C. Cao , P. Wu , X. Huang , D. Ma , Cancer Communications 2025, 45, 77. 39611440 10.1002/cac2.12629 PMC11833674

[39] C. Qian , R. Wang , H. Wu , F. Zhang , J. Wu , L. Wang , Anal. Chem. 2019, 91, 11362. 31403279 10.1021/acs.analchem.9b02554

[40] W. Guo , Y. Tao , R. Yang , K. Mao , H. Zhou , M. Xu , T. Sun , X. Li , C. Shi , Z. Ge , R. Xue , H. Zhou , Y. Ren , Science Advances 2024, 10, adq2899. 10.1126/sciadv.adq2899 PMC11559619 39536102

[41] Z. Chen , M. Schiffman , R. Herrero , R. DeSalle , K. Anastos , M. Segondy , V. V. Sahasrabuddhe , P. E. Gravitt , A. W. Hsing , R. D. Burk , PLoS One 2011, 6, 20183. 10.1371/journal.pone.0020183 PMC3103539 21673791

[42] J. Joung , A. Ladha , M. Saito , N.‐G. Kim , A. E. Woolley , M. Segel , R. P. J. Barretto , A. Ranu , R. K. Macrae , G. Faure , E. I. Ioannidi , R. N. Krajeski , R. Bruneau , M.‐L. W. Huang , X. G. Yu , J. Z. Li , B. D. Walker , D. T. Hung , A. L. Greninger , K. R. Jerome , J. S. Gootenberg , O. O. Abudayyeh , F. Zhang , N. Engl. J. Med. 2020, 383, 1492. 32937062 10.1056/NEJMc2026172 PMC7510942

