# Supplementary material for: A Field‐Deployable RotEx‐LAMP‐LFA Platform for Molecular Triage of HPV‐Driven Oncogenesis
Source: Adv Sci (Weinh). 2025 Sep 26;12(47):e09468. doi: 10.1002/advs.202509468 (PMC12713021; doi:10.1002/advs.202509468)
Supplement: Supplementary file 1 — Supporting Information [file ADVS-12-e09468-s001.pdf]

A Field-deployable RotEx-LAMP-LFA Platform for Molecular Triage of HPV-driven Oncogenesis

Yuan Gao, Qiang Wen, Siyuan Qiao, Linyi Deng, Keyue Li, Zhuyan Shao, Xiaonan Liu, Tao Zhu, Chao Zhang* Da Han* and Weihong Tan**

Y. Gao, S. Qiao, L. Deng, K. Li, C. Zhang, D. Han and W. Tan

Institute of Molecular Medicine (IMM)

Renji Hospital

School of Medicine

Shanghai Jiao Tong University

Shanghai 200240, China.

E-mail: chaozhang@sjtu.edu.cn; dahan@sjtu.edu.cn; tan@hnu.edu.cn

Q. Wen, Z. Shao, T Zhu, D. Han and W. Tan

Hangzhou Institute of Medicine (HIM)

Zhejiang Cancer Hospital

Chinese Academy of Sciences

Hangzhou, Zhejiang 310022 China

E-mail: zhutao@zjcc.org.cn

W. Tan

Molecular Science and Biomedicine Laboratory (MBL)

State Key Laboratory of Chemo/Biosensing and Chemometrics

College of Chemistry and Chemical Engineering

College of Biology

Aptamer Engineering Center of Hunan Province

Hunan University

Changsha, Hunan 410082, China

X. Liu

School of Forensic Medicine

Shanxi Medical University

Taiyuan, Shanxi 030001, China.

Figure S1. Photograph of the components used in the RotEx-LAMP-mLFA system: a) self-sampling swab and b) PTC heating plate.

a b

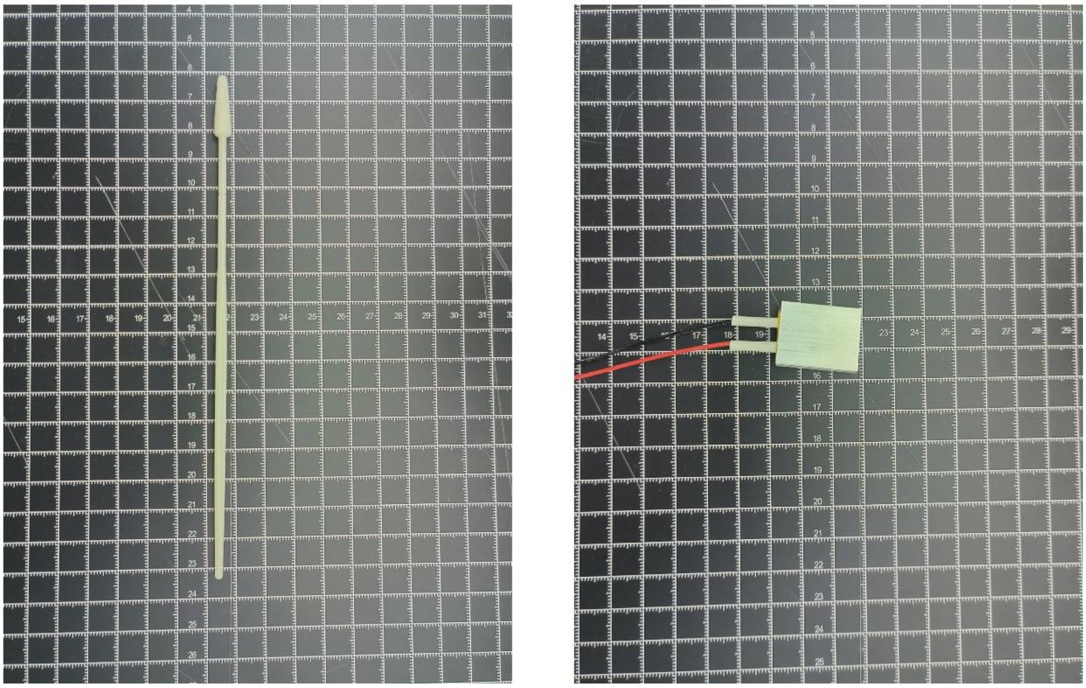

Figure S2. Schematic diagrams of the device components. a) Dimensional specifications of the device base; b) Design parameters of the rotatable extraction chamber; c) Structural layout of the reaction chip.

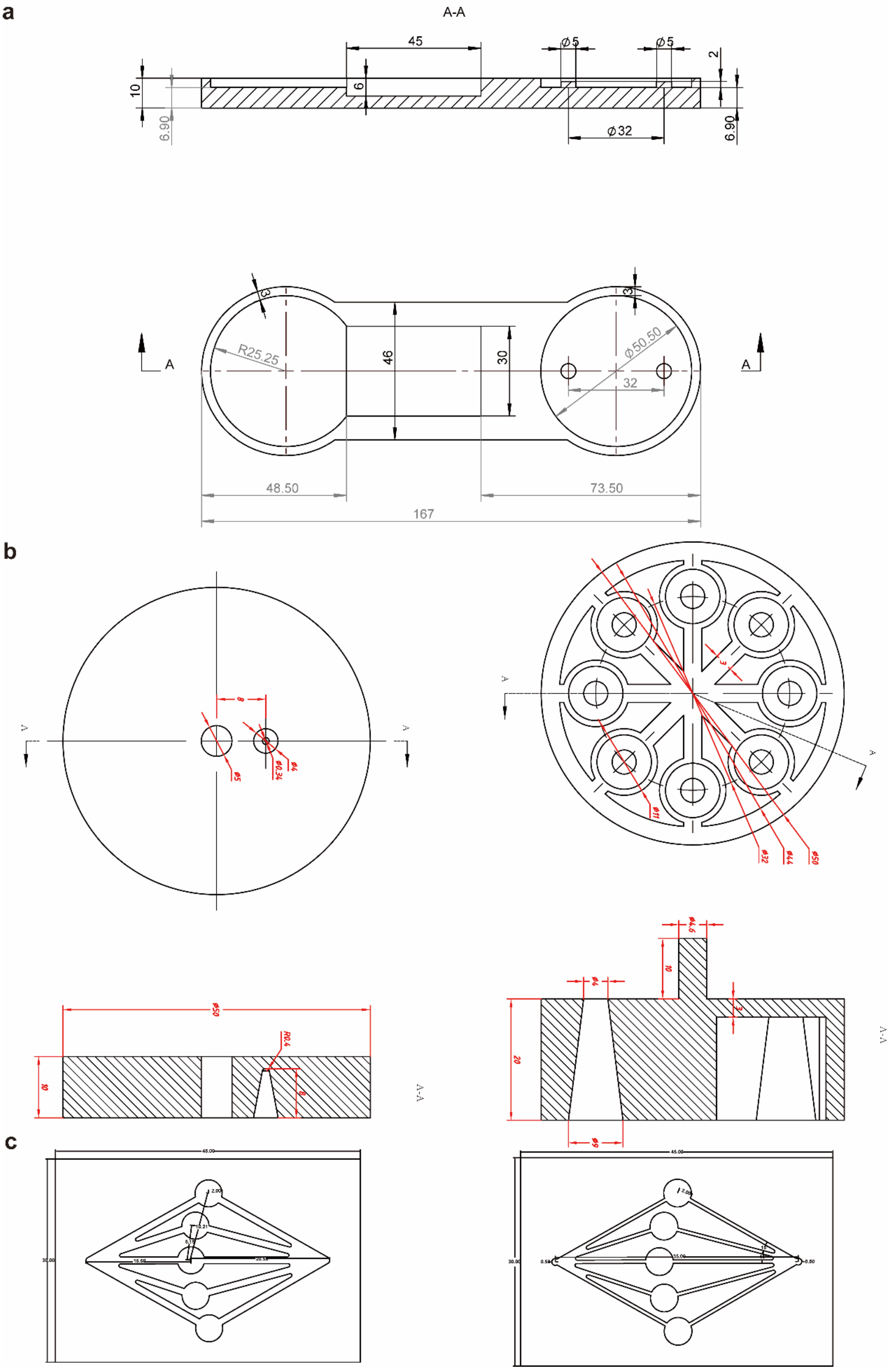

Figure S3. Performance validation and configuration details of the RotEx system.

a-b) The figure shows the effect of different shaking frequencies and durations on nucleic acid extraction efficiency. a) Shaking frequency and b) shaking time were precisely controlled using a laboratory shaker. Each test was conducted with 1×10^4 cells as input, and GAPDH was used as the internal reference gene for quantification. The frequency conversion relationship is: 60 rpm = 1 Hz. c) Radar chart comparing the average temperatures across five reaction chambers (P1-P5) in three units (Unit1-Unit3). For each unit, the average temperature was calculated over the timepoints at 10, 20, and 30 minutes. Each polygon represents one experimental repeat. The gray dashed line indicates the average temperature across replicates, while the gray shaded area represents the standard deviation range for each point. d) The diagram shows a top view of the RotEx rotatable extraction chamber. The left panel illustrates the top layer of the device; the middle panel displays the arrangement of reagents in each sector of the middle layer; the right panel indicates the placement of magnetic beads in the bottom layer. e) Operation time (in minutes) recorded for each volunteer across five experimental trials. f) Accuracy, sensitivity, and specificity of detection results for each volunteer. The metrics were calculated as follows: Accuracy = (TP + TN) / (TP + TN + FP + FN); Sensitivity (True Positive Rate) = TP / (TP + FN); Specificity (True Negative Rate) = TN / (TN + FP)

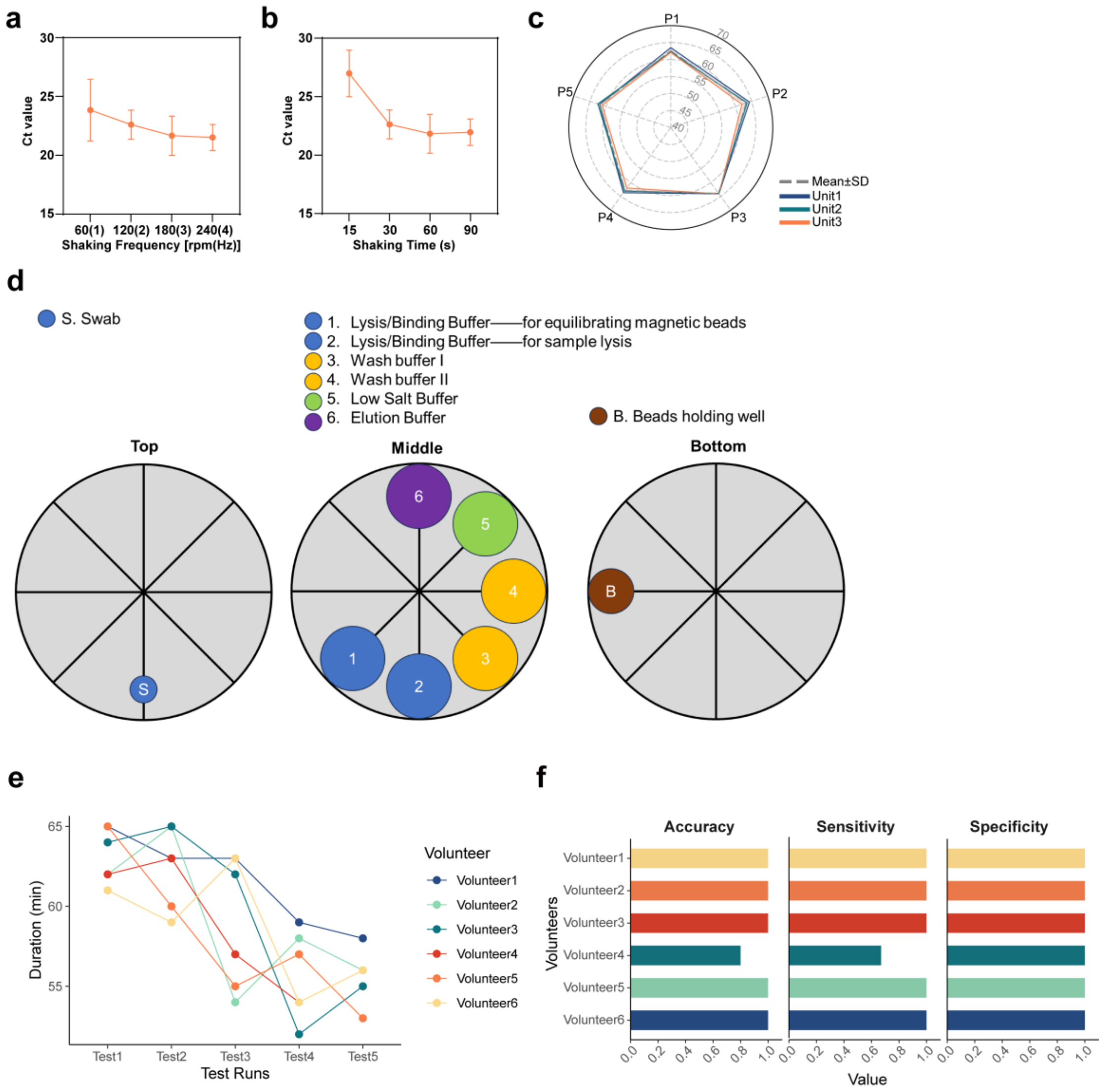

Figure S4. Comparison of RT-LAMP Amplification Efficiency with and without Biotin-dUTP. Fluorescence kinetic data were analyzed and are presented as the mean ± s.d. from three independent experiments (n = 3).

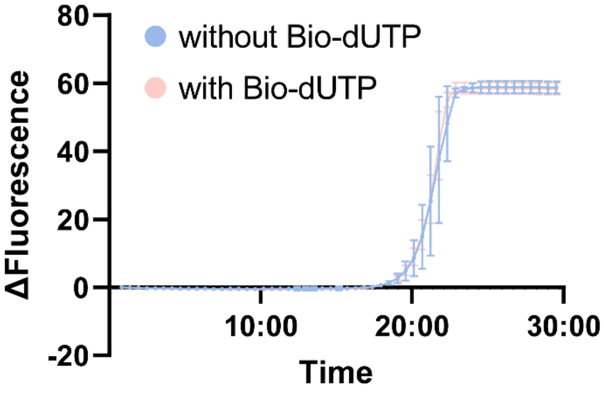

Figure S5. Fluorescence detection of HPV18 from synthetic plasmid (600 copies/μL) using different primer pairs (without loop primers) for E6 and E7 genes.

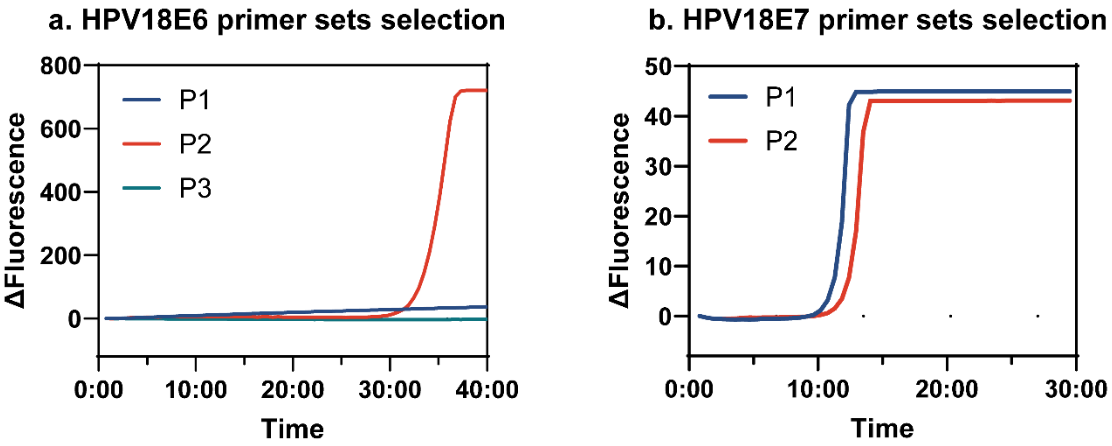

Figure S6. Fluorescence and gel electrophoresis analysis of HPV16 E6 and E7 from synthetic plasmid (600 copies/µL) using various primer concentrations.

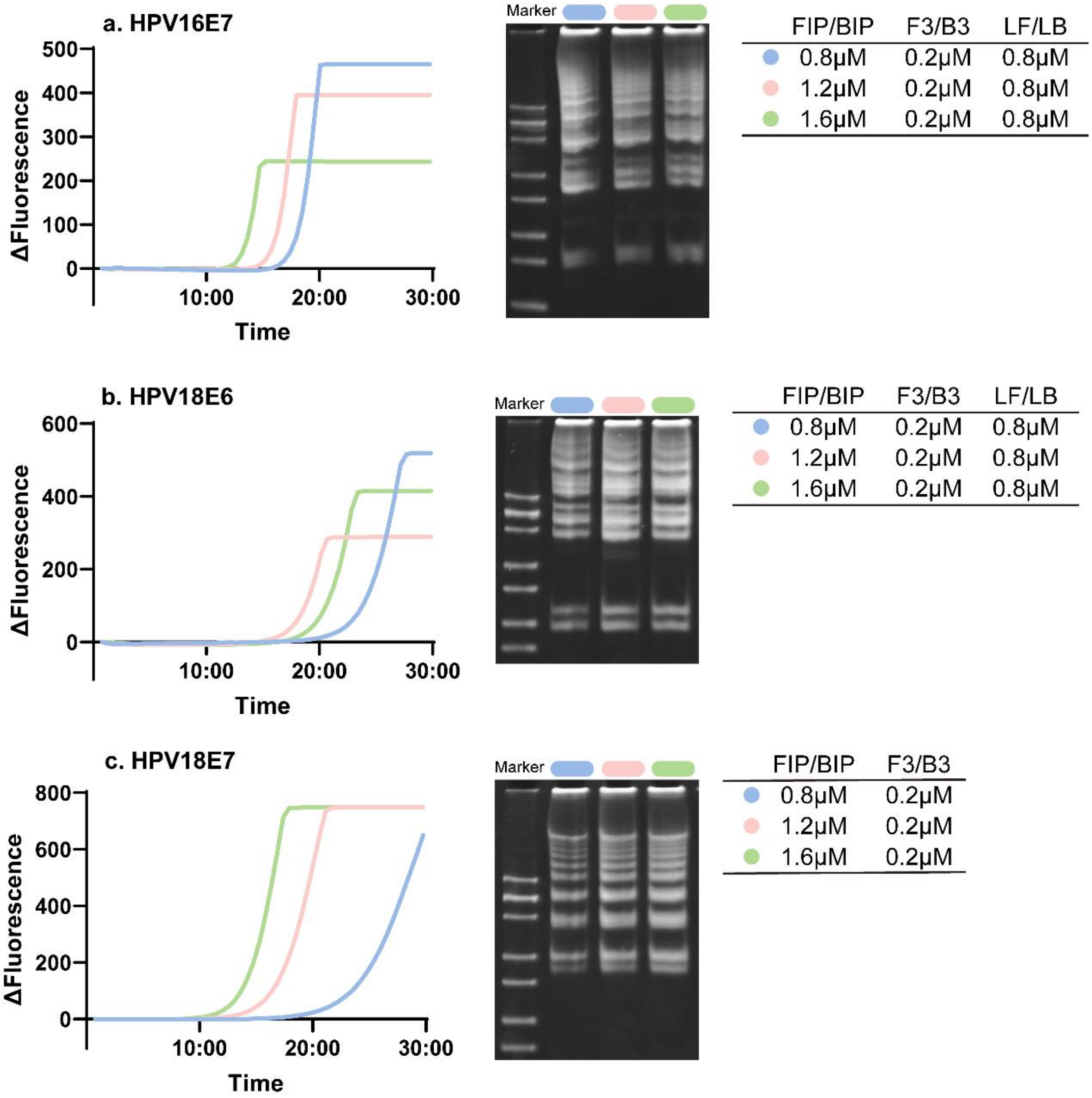

Figure S7. Fluorescence detection of different high-risk HPV types using the HPV16 primer set, with synthetic plasmid templates at 6000 copies/μL.

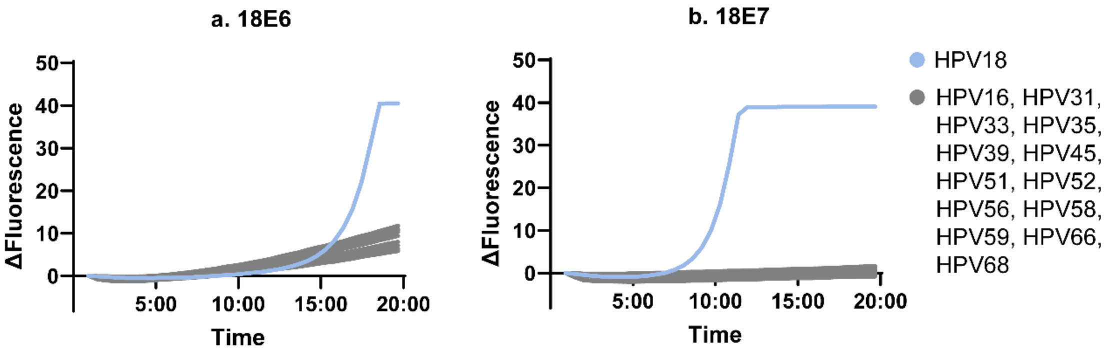

Figure S8. Gel electrophoresis analysis of HPV16 E7, HPV18 E6 and E7 from synthetic plasmid (60 copies/μL) using corresponding primer pairs under varying temperature conditions.

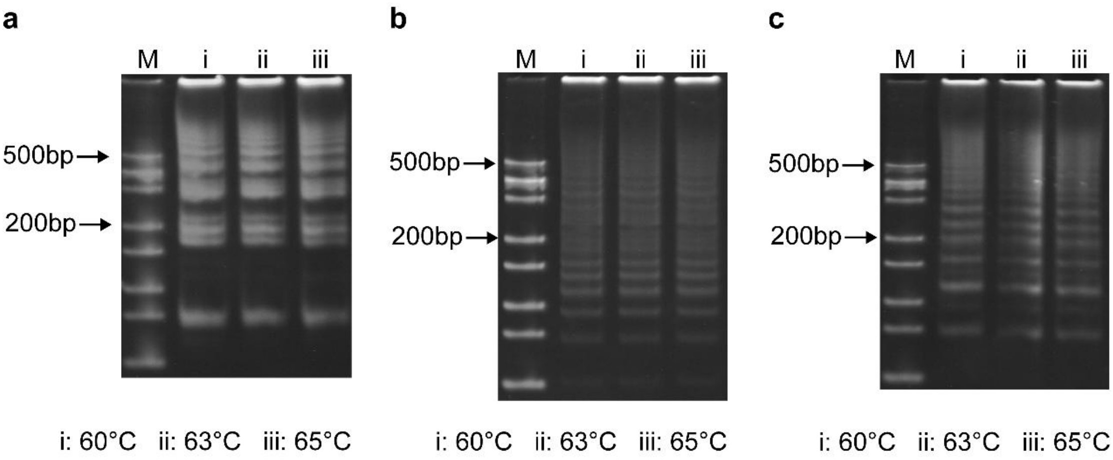

Figure S9. Principle of predicting cancer risk based on HPV mRNA and direct RT-LAMP amplification (left) and the structure and process of mLFA (right).

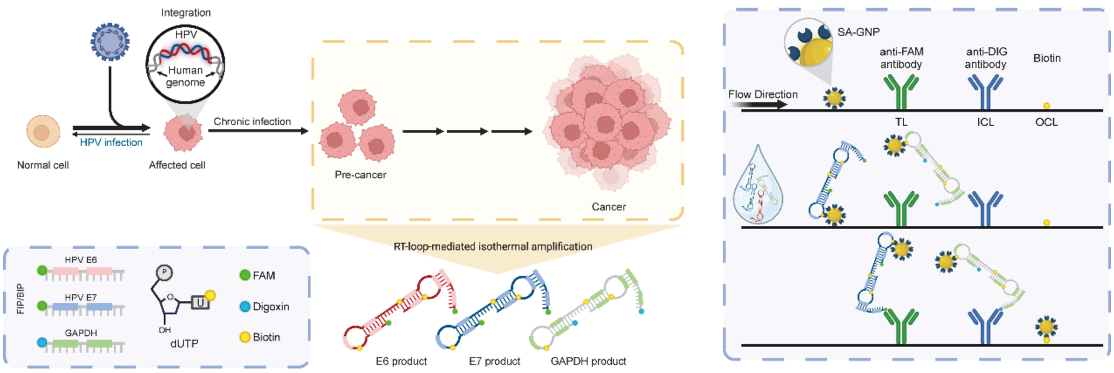

Figure S10. Dose-response relationship between HPV16/18 mRNA concentration and lateral flow signal intensity (0.24 – 750 copies/μL). The upper panel shows representative test strips after reaction; the lower panel presents the results of simple linear regression. The x-axis represents the log-transformed mRNA concentration, and the y-axis indicates the ratio of TL (test line) to OCL (outline control line) shadow intensity. The coefficient of determination (R^2) quantifies the goodness of fit.

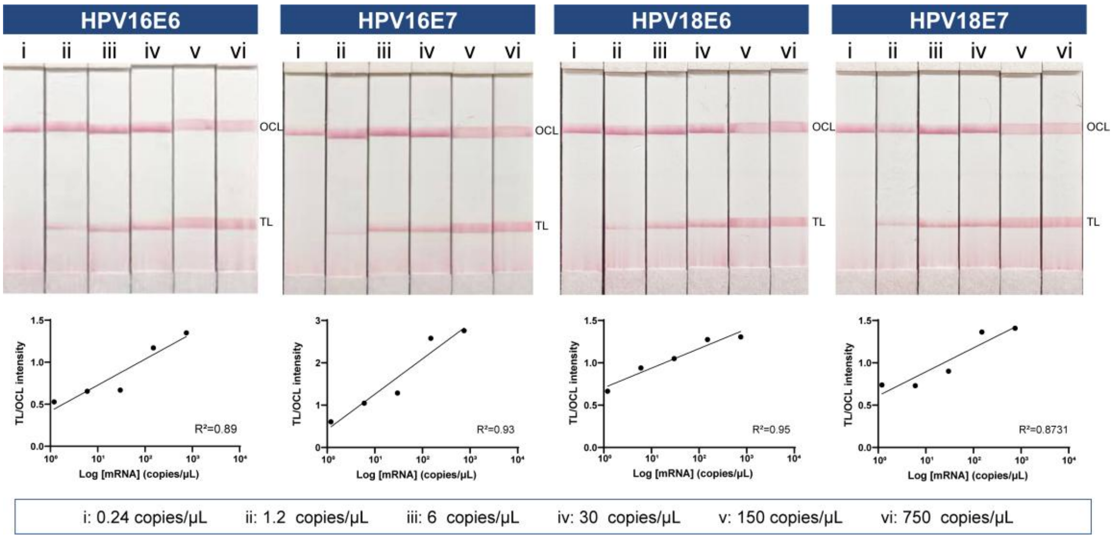

Figure S11. Diagnostic results of 14 types HR-HPV using the HPV18 primer sets. The template concentrations were 30 copies/μL for synthetic plasmids.

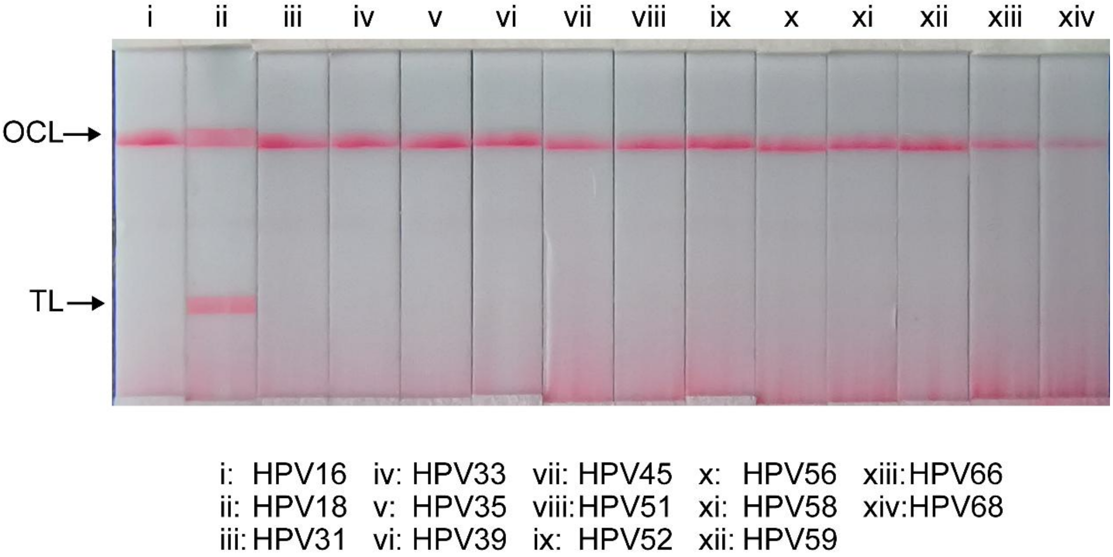

Table S1. Estimated Cost Range of Individual Components in the RotEx System.

**Component**	**Estimated Cost**
Disposable consumables and reagents	
PDMS chip fabrication	$1.5 - $3.0
Nucleic acid extraction reagents	$1.2 - $2.0
Amplification reagents (including enzymes, buffer)	$1.0 - $2.0
Primers (per reaction)	$0.1 - $0.2
Disposable nucleic acid lateral flow strip (LFA)	$0.4 - $1.3
Reusable components	
3D-printed device housing	$3.0 - $3.5
PTC heating element (Positive Temperature Coefficient resistor)	$1.0
Rechargeable lithium battery	$1.8 - $2.0

Table S2. Judgment results from the user panel, where 1 indicates a positive result and 0 indicates a negative result.

Participant	1	2	3	4	5	6	7	8	9	10
**HPV16E6**										
**0rep1**	0	0	0	0	0	0	0	0	0	0
**0rep2**	0	0	0	0	0	0	0	0	0	0
**0rep3**	0	0	0	0	0	0	0	0	0	0
**0.06rep1**	0	0	0	0	0	0	0	0	0	0
**0.06rep2**	0	0	0	0	0	0	0	0	0	0
**0.06rep3**	0	0	0	0	0	0	0	0	0	0
**0.6rep1**	1	0	1	1	0	0	0	1	0	0
**0.6rep2**	1	0	1	0	0	1	0	1	0	1
**0.6rep3**	1	0	1	0	0	1	0	0	0	1
**6rep1**	1	1	1	1	1	1	1	1	0	1
**6rep2**	1	1	1	1	1	1	1	1	1	1
**6rep3**	1	1	1	1	1	1	1	1	1	1
**60rep1**	1	1	1	1	1	1	1	1	1	1
**60rep2**	1	1	1	1	1	1	1	1	1	1
**60rep3**	1	1	1	1	1	1	1	1	1	1
**600rep1**	1	1	1	1	1	1	1	1	1	1
**600rep2**	1	1	1	1	1	1	1	1	1	1
**600rep3**	1	1	1	1	1	1	1	1	1	1
**HPV16E7**										
**0rep1**	0	0	0	0	0	0	0	0	0	0
**0rep2**	0	0	0	0	0	0	0	0	0	0
**0rep3**	0	0	0	0	0	0	0	0	0	0
**0.06rep1**	0	0	0	0	0	0	0	0	0	0
**0.06rep2**	0	0	0	0	0	0	0	0	0	0
**0.06rep3**	0	0	0	0	0	0	0	0	0	0
**0.6rep1**	1	1	1	1	0	0	1	1	0	0
**0.6rep2**	1	1	1	0	1	1	1	1	0	1
**0.6rep3**	1	0	1	0	0	1	0	0	0	1
**6rep1**	1	1	1	1	1	1	1	1	1	1
**6rep2**	1	1	1	1	1	1	1	1	1	1
**6rep3**	1	1	1	1	1	1	1	1	1	1
**60rep1**	1	1	1	1	1	1	1	1	1	1
**60rep2**	1	1	1	1	1	1	1	1	1	1
**60rep3**	1	1	1	1	1	1	1	1	1	1
**600rep1**	1	1	1	1	1	1	1	1	1	1
**600rep2**	1	1	1	1	1	1	1	1	1	1
**600rep3**	1	1	1	1	1	1	1	1	1	1
**HPV18E6**										
**0rep1**	0	0	0	0	0	0	0	0	0	0
**0rep2**	0	0	0	0	0	0	0	0	0	0

**0rep3**	0	0	0	0	0	0	0	0	0	0
**0.06rep1**	0	0	0	0	0	0	0	0	0	0
**0.06rep2**	0	0	0	0	0	0	0	0	0	0
**0.06rep3**	0	0	0	0	0	0	0	0	0	0
**0.6rep1**	1	1	1	1	0	0	1	1	0	0
**0.6rep2**	1	0	1	0	0	0	1	0	0	0
**0.6rep3**	1	0	1	0	0	0	0	0	0	1
**6rep1**	1	1	1	1	1	1	1	1	1	1
**6rep2**	1	1	1	1	1	1	1	1	1	1
**6rep3**	1	1	1	1	1	1	1	1	1	1
**60rep1**	1	1	1	1	1	1	1	1	1	1
**60rep2**	1	1	1	1	1	1	1	1	1	1
**60rep3**	1	1	1	1	1	1	1	1	1	1
**600rep1**	1	1	1	1	1	1	1	1	1	1
**600rep2**	1	1	1	1	1	1	1	1	1	1
**600rep3**	1	1	1	1	1	1	1	1	1	1
**HPV18E7**										
**0rep1**	0	0	0	0	0	0	0	0	0	0
**0rep2**	0	0	0	0	0	0	0	0	0	0
**0rep3**	0	0	0	0	0	0	0	0	0	0
**0.06rep1**	0	0	0	0	0	0	0	0	0	0
**0.06rep2**	0	0	0	0	0	0	0	0	0	0
**0.06rep3**	0	0	0	0	0	0	0	0	0	0
**0.6rep1**	1	1	1	1	0	0	1	1	0	1
**0.6rep2**	1	0	1	0	0	0	1	0	0	1
**0.6rep3**	1	0	1	0	0	1	0	1	0	1
**6rep1**	1	1	1	1	1	1	1	1	1	1
**6rep2**	1	1	1	1	1	1	1	1	1	1
**6rep3**	1	1	1	1	1	1	1	1	1	1
**60rep1**	1	1	1	1	1	1	1	1	1	1
**60rep2**	1	1	1	1	1	1	1	1	1	1
**60rep3**	1	1	1	1	1	1	1	1	1	1
**600rep1**	1	1	1	1	1	1	1	1	1	1
**600rep2**	1	1	1	1	1	1	1	1	1	1
**600rep3**	1	1	1	1	1	1	1	1	1	1

Table S3. RT-LAMP with specifically designed primers

Primer Set	Oligos	Sequence（5'→3'）
**16E6P1**	F3	ATGCACCAAAAGAGAACTG
	B3	AGCATATGGATTCCCATCTC
	FIP	GCAGCTCTGTGCATAACTGTCAATGTTTCAGGACCCACA
	BIP	AGAATGTGTGTACTGCAAGCAATCCCGAAAAGCAAAGTCAT
	LF	GGTAACTTTCTGGGTCGCTCC
	LB	CAGTTACTGCGACGTGAGGT
**16E6P2**	F3	GGAACAACATTAGAACAGCA
	B3	CGTGTTCTTGATGATCTGCA
	FIP	ACACAGTGGCTTTTGACAGTTATACAACAAACCGTTGTGTGA
	BIP	CTGAAGAAAAGCAAAGACATCTGGACAAGACATACATCGACCG
**16E7P1**	F3	GATACACCTACATTGCATGAA
	B3	CAACCGAAGCGTAGAGTC
	FIP	TCCTCTGAGCTGTCATTTAATTGCTAGATTTGCAACCAGAGACA
	BIP	GAGGATGAAATAGATGGTCCAGCACACTTGCAACAAAAGGTTAC
**16E7P2**	F3	CATGGAGATACACCTACATTG
	B3	CTTTGTACGCACAACCGA
	FIP	CTCTGAGCTGTCATTTAATTGCTCAGAATATATGTTAGATTTGCAACCAG
	BIP	GGACAAGCAGAACCGGACAGAGCGTAGAGTCACACTTG
	LF	ACAGTAGAGATCAGTTGTC
	LB	GCCCATTACAATATTGTAACC
**16E7P3**	F3	GATACACCTACATTGCATGAA
	B3	TTGTACGCACAACCGAAG
	FIP	ATCCTCCTCCTCTGAGCTGTCACCAGAGACAACTGATCTC
	BIP	TAGATGGTCCAGCTGGACAAGTAGAGTCACACTTGCAACAA
**16E7P4**	F3	AGACAACTGATCTCTACTGTT
	B3	CTTCCAAAGTACGAATGTCTAC
	FIP	TTCTGCTTGTCCAGCTGGACGCAATTAAATGACAGCTCAGAG
	BIP	CCGGACAGAGCCCATTACAATGTGTGTGCTTTGTACGCA
**16E7P5**	F3	AGATTTGCAACCAGAGACA
	B3	CTTTGTACGCACAACCGA
	FIP	ACCATCTATTTCATCCTCCTCCTACTGATCTCTACTGTTATGAGC
	BIP	GGACAAGCAGAACCGGACAGAGCGTAGAGTCACACTTG
**18E6P1**	F3	AAAAACTAACTAACACTGGGTTA
	B3	ACTTGTGTTTCTCTGCGT
	FIP	GGTGTCTAAGTTTTTCTGCTGGATAATTTATTAATAAGGTGCCTGCG
	BIP	CGACGATTTCACAACATAGCTGGGTTGGAGTCGTTCCTGTC
**18E6P2**	F3	ACAAGCTACCTGATCTGTG

	B3	ATCGTCGTTTTTCATTAAGGT
	FIP	CGGTATACTGTCTCTATACACCACACACGGAACTGAACACTTCA
	BIP	TATTCAGACTCTGTGTATGGAGACAAAGTTTTTCTGCTGGATTCAAC
	LP	CTGTAAGTTCCAATACTGTCTTGCA
	LB	TTTATTAATAAGGTGCCTGCGGTGC
**18E6P3**	F3	GAACACTTCACTGCAAGAC
	B3	CCCAGTGTTAGTTAGTTTTTCC
	FIP	CGGTATACTGTCTCTATACACCACAATATTGCAAGACAGTATTGGAAC
	BIP	CATGCTGCATGCCATAAATGTATAGATACACAGAGTCTGAATAATGTC
**18E7P1**	F3	CATTGCAAGACATTGTATTGC
	B3	GCTGAGCTTTCTACTACTAGC
	FIP	TCCTCTGAGTCGCTTAATTGCTGAGCCCCAAAATGAAATTCC
	BIP	ATCAACATTTACCAGCCCGACGTCAATTCTGGCTTCACACT
**18E7P2**	F3	TGAAATTCCGGTTGACCT
	B3	CTGGAATGCTCGAAGGTC
	FIP	CGGGCTGGTAAATGTTGATGATTAACACGAGCAATTAAGCGAC
	BIP	CCGAACCACAACGTCACACAACTAGCTCAATTCTGGCTT
**GAPDH**	F3	TCGTGGAAGGACTCATGACC
	B3	CGTTCAGCTCAGGGATGAC
	FIP	ACGCCACAGTTTCCCGGAGGACAGTCCATGCCATCACTGC
	BIP	CGCGGGGCTCTCCAGAACACTTGCCCACAGCCTTGG

Table S4. RT-qPCR primers sequences[6]

	FP	AATGTTTCAGGACCCACAGG
**HPV16E6**	RP	GTTGCTTGCAGTACACACATTC
	PB	ACCACAGTTATGCACAGAGCTGCA
	FP	TCAGAGGAGGAGGATGAAATAGA
**HPV16E7**	RP	GCACAACCGAAGCGTAGA
	PB	AGAACCGGACAGAGCCCATTACAA
	FP	ACCCTACAAGCTACCTGATCT
**HPV18E6**	RP	ACCTCTGTAAGTTCCAATACTGTC
	PB	ACGGAACTGAACACTTCACTGCAAGA
	FP	AATTCCGGTTGACCTTCTATGT
**HPV18E7**	RP	GGCTGGTAAATGTTGATGAT
	PB	TAAGCGACTCAGAGGAAGAAA
	FP	GGACCTGACCTGCCGTCTAG
**GAPDH**	RP	TAGCCCAGGATGCCCTTGAG
	PB	CCTCCGACGCCTGCTTCACCACCT

Table S5. Detailed sequences of synthetic pathogenic microorganism gene fragments.

Gene	Sequence（5'→3'）
**HPV16E6**	ATGTTTCAGGACCCACAGGAGCGACCCAGAAAGTTACCACAGTTATGCA<br>CAGAGCTGCAAACAACTATACATGATATAATATTAGAATGTGTGTACTG<br>CAAGCAACAGTTACTGCGACGTGAGGTATATGACTTTGCTTTTCGGGATT<br>TATGCATAGTATATAGAGATGGGAATCCATATGCTGTATGTGATAAATGT<br>TTAAAGTTTTATTCTAAAATTAGTGAGTATAGACATTATTGTTATAGTTT<br>GTATGGAACAACATTAGAACAGCAATACAACAAACCGTTGTGTGATTTG<br>TTAATTAGGTGTATTAACTGTCAAAAGCCACTGTGTCCTGAAGAAAAGC<br>AAAGACATCTGGACAAAAAGCAAAGATTCCATAATATAAGGGGTCGGTG<br>GACCGGTCGATGTATGTCTTGTTGCAGATCATCAAGAACACGTAGAGAA<br>ACCCAGCTGTAA
**HPV16E7**	ATGCATGGAGATACACCTACATTGCATGAATATATGTTAGATTTGCAACC<br>AGAGACAACTGATCTCTACTGTTATGAGCAATTAAATGACAGCTCAGAG<br>GAGGAGGATGAAATAGATGGTCCAGCTGGACAAGCAGAACCGGACAGA<br>GCCCATTACAATATTGTAACCTTTTGTTGCAAGTGTGACTCTACGCTTCG<br>GTTGTGCGTACAAAGCACACACGTAGACATTCGTACTTTGGAAGACCTG<br>TTAATGGGCACACTAGGAATTGTGTGCCCCATCTGTTCTCAGAAACCATA<br>A
**HPV18E6**	ATGGCGCGCTTTGAGGATCCAACACGGCGACCCTACAAGCTACCTGATC<br>TGTGCACGGAACTGAACACTTCACTGCAAGACATAGAAATAACCTGTGT<br>ATATTGCAAGACAGTATTGGAACTTACAGAGGTATTTGAATTTGCATTTA<br>AAGATTTATTTGTGGTGTATAGAGACAGTATACCGCATGCTGCATGCCAT<br>AAATGTATAGATTTTTATTCTAGAATTAGAGAATTAAGACATTATTCAGA<br>CTCTGTGTATGGAGACACATTGGAAAAACTAACTAACACTGGGTTATAC<br>AATTTATTAATAAGGTGCCTGCGGTGCCAGAAACCGTTGAATCCAGCAG<br>AAAAACTTAGACACCTTAATGAAAAACGACGATTTCACAACATAGCTGG<br>GCACTATAGAGGCCAGTGCCATTCGTGCTGCAACCGAGCACGACAGGAA<br>CGACTCCAACGACGCAGAGAAACACAAGTATAA
**HPV18E7**	ATGCATGGACCTAAGGCAACATTGCAAGACATTGTATTGCATTTAGAGC<br>CCCAAAATGAAATTCCGGTTGACCTTCTATGTCACGAGCAATTAAGCGA<br>CTCAGAGGAAGAAAACGATGAAATAGATGGAGTTAATCATCAACATTTA<br>CCAGCCCGACGAGCCGAACCACAACGTCACACAATGTTGTGTATGTGTT<br>GTAAGTGTGAAGCCAGAATTGAGCTAGTAGTAGAAAGCTCAGCAGACGA<br>CCTTCGAGCATTCCAGCAGCTGTTTCTGAACACCCTGTCCTTTGTGTGTCC<br>GTGGTGTGCATCCCAGCAGTAA
**HPV31E6**	ATGTTCAAAAATCCTGCAGAAAGACCTCGGAAATTGCATGAACTAAGCT<br>CGGCATTGGAAATACCCTACGATGAACTAAGATTGAATTGTGTCTACTGC<br>AAAGGTCAGTTAACAGAAACAGAGGTATTAGATTTTGCATTTACAGATT<br>TAACAATAGTATATAGGGACGACACACCACACGGAGTGTGTACAAAATG<br>TTTAAGATTTTATTCAAAAGTAAGTGAATTTAGATGGTATAGATATAGTG<br>TGTATGGAACAACATTAGAAAAATTGACAAACAAAGGTATATGTGATTT<br>GTTAATTAGGTGTATAACGTGTCAAAGACCGTTGTGTCCAGAAGAAAAA<br>CAAAGACATTTGGATAAAAAGAAACGATTCCACAACATAGGAGGAAGG

	TGGACAGGACGTTGCATAGCATGTTGGAGAAGACCTCGTACTGAAACCC<br>AAGTGTAA
**HPV31E7**	ATGCGTGGAGAAACACCTACGTTGCAAGACTATGTGTTAGATTTGCAAC<br>CTGAGGCAACTGACCTCCACTGTTATGAGCAATTACCCGACAGCTCAGA<br>TGAGGAGGATGTCATAGACAGTCCAGCTGGACAAGCAGAACCGGACAC<br>ATCCAATTACAATATCGTTACCTTTTGTTGTCAGTGTAAGTCTACACTTCG<br>TTTGTGTGTACAGAGCACACAAGTAGATATTCGCATATTGCAAGAGCTGT<br>TAATGGGCTCATTTGGAATCGTGTGCCCCAACTGTTCTACTAGACTGTAA
**HPV33E6**	ATGTTTCAAGACACTGAGGAAAAACCACGAACATTGCATGATTTGTGCC<br>AAGCATTGGAGACAACTATACACAACATTGAACTACAGTGCGTGGAATG<br>CAAAAAACCTTTGCAACGATCTGAGGTATATGATTTTGCATTTGCAGATT<br>TAACAGTTGTATATAGAGAGGGAAATCCATTTGGAATATGTAAACTGTG<br>TTTGCGGTTCTTATCTAAAATTAGTGAATATAGACATTATAATTATTCTGT<br>ATATGGAAATACATTAGAACAAACAGTTAAAAAACCTTTAAATGAAATA<br>TTAATTAGGTGTATTATATGTCAAAGACCTTTGTGTCCTCAAGAAAAAAA<br>ACGACATGTGGATTTAAACAAACGATTTCATAATATTTCGGGTCGTTGGG<br>CAGGGCGCTGTGCGGCGTGTTGGAGGTCCCGACGTAGAGAAACTGCACT<br>GTGA
**HPV33E7**	ATGAGAGGACACAAGCCAACGTTAAAGGAATATGTTTTAGATTTATATC<br>CTGAACCAACTGACCTATACTGCTATGAGCAATTAAGTGACAGCTCAGA<br>TGAGGATGAAGGCTTGGACCGGCCAGATGGACAAGCACAACCAGCCAC<br>AGCTGATTACTACATTGTAACCTGTTGTCACACTTGTAACACCACAGTTC<br>GTTTATGTGTCAACAGTACAGCAAGTGACCTACGAACCATACAGCAACT<br>ACTTATGGGCACAGTGAATATTGTGTGCCCTACCTGTGCACAACAATAA
**HPV35E6**	ATGTTTCAGGACCCAGCTGAACGACCTTACAAACTGCATGATTTGTGCAA<br>CGAGGTAGAAGAAAGCATCCATGAAATTTGTTTGAATTGTGTATACTGC<br>AAACAAGAATTACAGCGGAGTGAGGTATATGACTTTGCATGCTATGATT<br>TGTGTATAGTATATAGAGAAGGCCAGCCATATGGAGTATGCATGAAATG<br>TTTAAAATTTTATTCAAAAATAAGTGAATATAGATGGTATAGATATAGTG<br>TGTATGGAGAAACGTTAGAAAAACAATGCAACAAACAGTTATGTCATTT<br>ATTAATTAGGTGTATTACATGTCAAAAACCGCTGTGTCCAGTTGAAAAGC<br>AAAGACATTTAGAAGAAAAAAAACGATTCCATAACATCGGTGGACGGTG<br>GACAGGTCGGTGTATGTCCTGTTGGAAACCAACACGTAGAGAAACCGAG<br>GTGTAA
**HPV35E7**	ATGCATGGAGAAATAACTACATTGCAAGACTATGTTTTAGATTTGGAAC<br>CCGAGGCAACTGACCTATACTGTTATGAGCAATTGTGTGACAGCTCAGA<br>GGAGGAGGAAGATACTATTGACGGTCCAGCTGGACAAGCAAAACCAGA<br>CACCTCCAATTATAATATTGTAACGTCCTGTTGTAAATGTGAGGCGACAC<br>TACGTCTGTGTGTACAGAGCACACACATTGACATACGTAAATTGGAAGA<br>TTTATTAATGGGCACATTTGGAATAGTGTGCCCCGGCTGTTCACAGAGAG<br>CATAA

**HPV39E6**	ATGGCGCGATTTCACAATCCTGCAGAACGGCCATACAAATTGCCAGACC TGTGCACAACGCTGGACACCACCTTGCAGGACATTACAATAGCCTGTGT CTATTGCAGACGACCACTACAGCAAACCGAGGTATATGAATTTGCATTT AGTGATTTATATGTAGTATATAGGGACGGGGAACCACTAGCTGCATGCC AATCATGTATAAAATTTTATGCTAAAATACGGGAGCTACGATATTACTCG GACTCGGTGTATGCAACTACATTAGAAAATATAACTAATACAAAGTTAT ATAATTTATTAATAAGGTGCATGTGTTGTCTGAAACCGCTGTGTCCAGCA GAAAAATTAAGACACCTAAATAGCAAACGAAGATTTCATAAAATAGCAG GAAGCTATACAGGACAGTGTCGACGGTGCTGGACCACAAAACGGGAGG ACCGCAGACTAACACGAAGAGAAACCCAAGTATAA
**HPV39E7**	ATGCGTGGACCAAAGCCCACCTTGCAGGAAATTGTATTAGATTTATGTCC TTACAATGAAATACAGCCGGTTGACCTTGTATGTCACGAGCAATTAGGA GAGTCAGAGGATGAAATAGATGAACCCGACCATGCAGTTAATCACCAAC ATCAACTACTAGCCAGACGGGATGAACCACAGCGTCACACAATACAGTG TTCGTGTTGTAAGTGTAACAACACACTGCAGCTGGTAGTAGAAGCCTCA CGGGATACTCTGCGACAACTACAGCAGCTGTTTATGGACTCACTAGGATT TGTGTGTCCGTGGTGTGCAACTGCAAACCAGTAA
**HPV45E6**	ATGGCGCGCTTTGACGATCCAAAGCAACGACCCTACAAGCTACCAGATT TGTGCACAGAATTGAATACATCACTACAAGACGTATCTATTGCCTGTGTA TATTGCAAAGCAACATTGGAACGCACAGAGGTATATCAATTTGCTTTTAA AGATTTATGTATAGTGTATAGAGACTGTATAGCATATGCTGCATGCCATA AATGTATAGACTTTTATTCCAGAATTAGAGAATTAAGATATTATTCAAAC TCTGTATATGGAGAGACACTGGAAAAAATAACTAATACAGAGTTGTATA ATTTGTTAATAAGGTGCCTGCGGTGCCAGAAACCATTGAACCCAGCAGA AAAACGTAGACACCTTAAGGACAAACGAAGATTTCACAGCATAGCTGGA CAGTACCGAGGGCAGTGTAATACATGTTGTGACCAGGCACGGCAAGAAA GACTTCGCAGACGTAGGGAAACACAAGTATAG
**HPV45E7**	ATGCATGGACCCCGGGAAACACTGCAAGAAATTGTATTGCATTTGGAAC CTCAGAATGAATTAGATCCTGTTGACCTGTTGTGTTACGAGCAATTAAGC GAGTCAGAGGAGGAAAACGATGAAGCAGATGGAGTTAGTCATGCACAA CTACCAGCCCGACGAGCCGAACCACAGCGTCACAAAATTTGTGTGTAT GTTGTAAGTGTGACGGCAGAATTGAGCTTACAGTAGAGAGCTCGGCAGA GGACCTTAGAACACTACAGCAGCTGTTTTTGAGCACCTTGTCCTTTGTGT GTCCGTGGTGTGCAACTAACCAATAA
**HPV51E6**	ATGTTCGAAGACAAGAGGGAAAGACCACGAACGCTGCATGAATTATGTG AAGCTTTGAACGTTTCTATGCACAATATACAGGTAGTGTGTGTGTATTGT AAAAAGGAATTATGTAGAGCAGATGTATATAATGTAGCATTTACTGAAA TTAAGATTGTATATAGGGATAATAATCCATATGCAGTATGCAAACAATG TTTACTGTTTTATTCAAAAATTAGAGAGTATAGACGTTATAGCAGGTCTG TGTATGGTACTACATTAGAGGCAATTACTAAAAAAAGCTTATATGATTTA TCGATAAGGTGTCATAGATGTCAAAGACCACTTGGGCCTGAAGAAAAGC AAAAATTGGTGGACGAAAAAAAAAGGTTCCATGAAATAGCGGGACGTT GGACGGGGCAATGCGCTAATTGCTGGCAACGTACACGACAACGTAACGA AACCCAAGTGTAA

**HPV51E7**	ATGCGTGGTAATGTACCACAATTAAAAGATGTAGTATTGCATTTAACACC<br>ACAGACTGAAATTGACTTGCAATGCTACGAGCAATTTGACAGCTCAGAG<br>GAGGAGGATGAAGTAGATAATATGCGTGACCAGCTACCAGAAAGACGG<br>GCTGGACAGGCTACGTGTTACAGAATTGAAGCTCCGTGTTGCAGGTGTTC<br>AAGTGTAGTACAACTGGCAGTGGAAAGCAGTGGAGACACCCTTCGCGTT<br>GTACAGCAGATGTTAATGGGCGAACTAAGCCTGGTTTGCCCGTGTTGTGC<br>GAACAACTAG
**HPV52E6**	ATGTTTGAGGATCCAGCAACACGACCCCGGACCCTGCACGAATTGTGTG<br>AGGTGCTGGAAGAATCGGTGCATGAAATAAGGCTGCAGTGTGTGCAGTG<br>CAAAAAAGAGCTACAACGAAGAGAGGTATACAAGTTTCTATTTACAGAT<br>TTACGAATAGTATATAGAGACAATAATCCATATGGCGTGTGTATTATGTG<br>CCTACGCTTTTTATCTAAGATAAGTGAATATAGGCATTATCAATATTCAC<br>TGTATGGGAAAACATTAGAAGAGAGGGTAAAAAAACCATTAAGTGAAA<br>TAACTATTAGATGTATAATTTGTCAAACGCCATTATGTCCTGAAGAAAAA<br>GAAAGACATGTTAATGCAAACAAGCGATTTCATAATATTATGGGTCGTT<br>GGACAGGGCGCTGTTCAGAGTGTTGGAGACCCCGACCTGTGACCCAAGT<br>GTAA
**HPV52E7**	ATGCGTGGAGACAAAGCAACTATAAAAGATTATATATTAGATCTGCAAC<br>CTGAAACAACTGACCTACACTGCTATGAGCAATTAGGTGACAGCTCAGA<br>TGAGGAGGATACAGATGGTGTGGACCGGCCAGATGGACAAGCAGAACA<br>AGCCACAAGCAATTACTACATTGTGACATATTGTCACAGTTGTGATAGCA<br>CACTACGGCTATGCATTCATAGCACTGCGACGGACCTTCGTACTCTACAG<br>CAAATGCTGTTGGGCACATTACAAGTTGTGTGCCCCGGCTGTGCACGGCT<br>ATAA
**HPV58E6**	ATGTTCCAGGACGCAGAGGAGAAACCACGGACATTGCATGATTTGTGTC<br>AGGCGTTGGAGACATCTGTGCATGAAATCGAATTGAAATGCGTTGAATG<br>CAAAAAGACTTTGCAGCGATCTGAGGTATATGACTTTGTATTTGCAGATT<br>TAAGAATAGTGTATAGAGATGGAAATCCATTTGCAGTATGTAAAGTGTG<br>CTTACGATTGCTATCTAAAATAAGTGAGTATAGACATTATAATTATTCGC<br>TATATGGAGACACATTAGAACAAACACTAAAAAAGTGTTTAAATGAAAT<br>ATTAATTAGATGTATTATTTGTCAAAGACCATTGTGTCCACAAGAAAAAA<br>AAAGGCATGTGGATTTAAACAAAAGGTTTCATAATATTTCGGGTCGTTG<br>GACAGGGCGCTGTGCAGTGTGTTGGAGACCCCGACGTAGACAAACACAA<br>GTGTAA
**HPV58E7**	ATGAGAGGAAACAACCCAACGCTAAGAGAATATATTTTAGATTTACATC<br>CTGAACCAACTGACCTATTCTGCTATGAGCAATTATGTGACAGCTCAGAC<br>GAGGATGAAATAGGCTTGGACGGGCCAGATGGACAAGCACAACCGGCC<br>ACAGCTAATTACTACATTGTAACTTGTTGTTACACTTGTGGCACCACGGT<br>TCGTTTGTGTATCAACAGTACAACAACCGACGTACGAACCCTACAGCAG<br>CTGCTTATGGGCACATGTACCATTGTGTGCCCTAGCTGTGCACAGCAATA<br>A
**HPV56E6**	ATGGAGCCACAATTCAACAATCCACAGGAACGTCCACGAAGCCTGCACC<br>ACTTGAGTGAGGTATTAGAAATACCTTTAATTGATCTTAGATTATCATGT<br>GTATATTGCAAAAAAGAACTAACACGTGCTGAGGTATATAATTTTGCAT

	GCACTGAATTAAAATTAGTGTATAGGGATGATTTCCTTATGCAGTGTGC AGAGTATGTTTATTGTTTTATAGTAAAGTTAGAAAATATAGGTATTATGA CTATTCAGTGTATGGAGCTACACTAGAAAGTATAACTAAAAAACAGTTA TGTGATTTATTAATAAGGTGCTACAGATGTCAAAGTCCGTTAACTCCGGA GGAAAAGCAATTGCATTGTGACAGAAAAAGACGATTTCATCTAATAGCA CATGGTTGGACCGGGTCATGTTTGGGGTGCTGGAGACAAACATCTAGAG AACCTAGAGAATCTACAGTATAA
**HPV56E7**	ATGCATGGTAAAGTACCAACGCTGCAAGACGTTGTATTAGAACTAACAC CTCAAACAGAAATTGACCTACAGTGCAATGAGCAATTGGACAGCTCAGA GGATGAGGATGAGGATGAAGTAGACCATTTGCAGGAGCGGCCACAGCA AGCTAGACAAGCTAAACAACATACGTGTTACCTAATACACGTACCTTGTT GTGAGTGTAAGTTTGTGGTGCAGTTGGACATTCAGAGTACCAAAGAGGA CCTGCGTGTTGTACAACAGCTGCTTATGGGTGCGTTAACAGTAACGTGCC CACTCTGCGCATCAAGTAACTAA
**HPV59E6**	ATGGCACGCTTTGAGGATCCTACACAACGACCATACAAACTGCCTGATTT GAGCACAACATTGAATATTCCTCTGCATGATATTCGCATCAATTGTGTGT TTTGCAAAGGGGAACTGCAAGAAAGAGAGGTATTTGAATTTGCTTTTAA TGACTTATTTATAGTGTATAGAGACTGTACACCGTATGCAGCGTGTCTGA AATGCATTTCATTTTATGCAAGAGTAAGAGAATTAAGATATTATAGAGA TTCCGTGTATGGAGAAACATTAGAGGCTGAAACCAAGACACCGTTACAT GAGCTGCTGATACGCTGTTATAGATGCCTAAAACCTCTATGTCCAACAGA TAAATTAAAGCATATAACTGAAAAAAGAAGATTCCATAATATAGCTGGA ATATATACAGGACAGTGTCGTGGGTGTCGGACCCGAGCAAGACACCTAA GACAGCAACGACAAGCGCGTAGTGAAACACTGGTGTAA
**HPV59E7**	ATGCATGGACCAAAAGCAACACTTTGTGACATTGTTTTAGATTTGGAACC ACAAAATTATGAGGAAGTTGACCTTGTGTGCTACGAGCAATTACCTGAC TCCGACTCCGAGAATGAAAAAGATGAACCAGATGGAGTTAATCATCCTT TGCTACTAGCTAGACGAGCTGAACCACAGCGTCACAACATTGTGTGTGT GTGTTGTAAGTGTAATAATCAACTTCAGCTAGTAGTAGAAACCTCGCAA GACGGATTGCGAGCCTTACAGCAGCTGTTTATGGACACACTATCCTTTGT GTGTCCTTTGTGTGCAGCAAACCAGTAA
**HPV66E6**	ATGGATTCCATATTCAGCAATACACAGGAACGTCCACGAAGCCTGCACC ATCTGAGCGAGGTATTACAAATACCTTTACTTGATCTTAGATTATCATGT GTATACTGCAAAAAGGAACTTACAAGTTTAGAGCTATATAGGTTTGCAT GTATTGAGTTAAAACTAGTATATAGAAACAATTGGCCATATGCAGTATG TAGGGTATGTTTATTGTTTTATAGTAAGGTTAGAAAATATAGGTACTATA AATATTCAGTGTATGGGGCAACATTAGAAAGTATAACTAAAAAACAGTT ATCTGATTTATCAATAAGGTGCTACCGATGTCAATGTCCGTTAACACCGG AGGAAAAACAATTGCACTGTGAACATAAAAGACGATTTCATTATATAGC ATATGCATGGACCGGGTCATGTTTGCAGTGTTGGAGACATACGAGTAGA CAAGCTACAGAATCTACAGTATAA
**HPV66E7**	ATGCATGGTAAAGTACCAACGTTGCAAGAGGTTATATTAGAACTTGCAC CGCAAACGGAAATTGACCTACAATGCAATGAGCAATTGGACAGCTCAGA GGATGAGGATGAGGATGAAATAGACCATTTGCTGGAGCGGCCACAGCA

	AGCTAGACAAGCTGAACAACATAAGTGTTACCTAATTCACGTACCTTGTT<br>GTAAGTGTGAGTTGGTGGTGCAGTTGGACATTCAGAGTACCAAAGAGGA<br>GCTACGTGTGGTACAACAGCTGCTTATGGGTGCGTTAACAGTAACGTGC<br>CCACTCTGCGCATCATCTAAATAA
**HPV68E6**	ATGGCGCTATTTCACAACCCTGAGGAACGGCCATACAAATTGCCAGACC<br>TGTGCAGGACATTGGACACTACATTGCATGACGTTACAATAGACTGTGTC<br>TATTGCAGAAGGCAACTACAACGGACAGAGGTATATGAATTTGCCTTTA<br>GTGACCTATGTGTAGTGTATAGAGACGGGGTACCATTTGCTGCATGCCA<br>ATCATGTATTAAATTTTATGCTAAAATACGGGAACTACGATATTACTCGG<br>AATCGGTGTATGCAACTACATTAGAAACCATAACTAATACAAAGTTATA<br>TAATTTATTGATAAGGTGCATGAGTTGCCTGAAACCATTGTGTCCAGCAG<br>AAAAACTAAGGCACCTAACAACAAAACGAAGATTACATAAAATAGCAG<br>GAAACTTTACAGGACAGTGTCGGCACTGCTGGACCAGTAAGCGAGAGGA<br>CCGCAGACGCATACGTCAAGAAACACAAGTTTAA
**HPV68E7**	ATGCATGGACCAAAGCCCACCGTGCAGGAAATTGTGTTAGAGCTATGTC<br>CATACAATGAAATACAGCCGGTTGACCTTGTATGTCACGAGCAATTAGG<br>AGATTCAGACGATGAAATAGATGAACCCGACCATGCAGTTAATCACCAC<br>CAACATCTACTACTAGCCAGACGGGACGAACAACAGCGTCACAGAATTC<br>AGTGTCTGTGTTGTAAGTGTAACAAGGCACTGCAACTAGTAGTAGAAGC<br>GTCGCGGGACAACCTGCGGACACTACAACAGCTGTTTATGGACTCACTA<br>AATTTTGTGTGTCCGTGGTGTGCAACTGAAACCCAGTAA

Table S6. Details of 69 clinical samples. Group 1 corresponds to the classification in Figure 5a, and Group 2 corresponds to the classification in Figure 5d.

Patient	Group1	Group2	Clinician-collected	Self-collected	RotEx-LAMP-LFA	RT-qPCR
P1	Cancer	Cancer	yes	no	+	+
P2	Cancer	Cancer	yes	no	+	+
P3	Cancer	Cancer	yes	no	+	+
P4	Cancer	Cancer	yes	no	+	+
P5	Cancer	Cancer	yes	no	+	+
P6	Infection	non-cacner	yes	no	-	-
P7	Pre-cancer	CIN2	yes	no	+	+
P8	Cancer	Cancer	yes	no	+	+
P9	Infection	non-cacner	yes	no	-	-
P10	Infection	non-cacner	yes	no	-	-
P11	Cancer	Cancer	yes	no	+	+
P12	Cancer	Cancer	yes	no	+	+
P13	Pre-cancer	CIN2	yes	no	+	+
P14	Cancer	Cancer	yes	no	+	+
P15	Cancer	Cancer	yes	no	+	+
P16	Pre-cancer	CIN2	yes	no	+	+
P17	Pre-cancer	CIN2	yes	no	-	+
P18	Infection	non-cacner	yes	no	-	-
P19	Infection	non-cacner	yes	no	-	-
P20	Infection	non-cacner	yes	no	-	-
P21	Infection	non-cacner	yes	no	-	-
P22	HC	non-cacner	yes	no	-	-
P23	HC	non-cacner	yes	no	-	-
P24	HC	non-cacner	yes	no	-	-
P25	HC	non-cacner	yes	no	-	-
P26	HC	non-cacner	yes	no	-	-
P27	HC	non-cacner	yes	no	-	-
P28	Pre-cancer	CIN1	yes	no	-	-
P29	Cancer	Cancer	yes	no	+	+
P30	Cancer	Cancer	yes	no	+	+
P31	Cancer	Cancer	yes	no	-	+
P32	HC	non-cacner	yes	no	-	-
P33	HC	non-cacner	yes	no	-	-
P34	Infection	non-cacner	yes	no	-	-

P35	Cancer	Cancer	yes	no	+	+
P36	HC	non-cacner	yes	no	-	-
P37	Cancer	Cancer	yes	no	+	+
P38	Pre-cancer	CIN1	yes	no	+	+
P39	HC	non-cacner	yes	no	-	-
P40	Cancer	Cancer	yes	no	+	+
P41	Cancer	Cancer	yes	no	+	+
P42	Cancer	Cancer	yes	no	+	+
P43	Pre-cancer	CIN2	yes	no	+	+
P44	Pre-cancer	CIN3	yes	no	+	+
P45	Pre-cancer	CIN2	yes	no	+	+
P46	Pre-cancer	CIN3	yes	no	+	+
P47	Pre-cancer	CIN3	yes	no	+	+
P48	Pre-cancer	CIN1	yes	no	-	-
P49	Infection	non-cacner	yes	no	-	-
P50	Infection	non-cacner	yes	no	-	-
P51	Infection	non-cacner	yes	no	-	-
P52	Infection	non-cacner	yes	no	-	-
P53	Infection	non-cacner	yes	no	-	-
P54	Infection	non-cacner	yes	no	-	-
P55	Infection	non-cacner	yes	no	-	-
P56	HC	non-cacner	yes	no	-	-
P57	HC	non-cacner	yes	no	-	-
P58	HC	non-cacner	yes	no	-	-
P59	HC	non-cacner	yes	no	-	-
P60	HC	non-cacner	yes	no	-	-
P61	HC	non-cacner	yes	no	-	-
P62	HC	non-cacner	yes	no	-	-
P63	HC	non-cacner	yes	no	-	-
P64	HC	non-cacner	yes	no	-	-
P65	HC	non-cacner	yes	no	-	-
P66	Cancer	Cancer	yes	yes	+	+
P67	Pre-cancer	CIN3	yes	yes	+	+
P68	Pre-cancer	CIN3	yes	yes	+	+

P69	HC	non-cacner	yes	yes	-	-

(1) Cook, D. A.; Mei, W.; Smith, L. W.; Van Niekerk, D. J.; Ceballos, K.; Franco, E. L.; Coldman, A. J.; Ogilvie, G. S.; Krajden, M. Comparison of the Roche Cobas® 4800 and Digene Hybrid Capture® 2 HPV Tests for Primary Cervical Cancer Screening in the HPV FOCAL Trial. *BMC Cancer* **2015**, *15* (1), 968. https://doi.org/10.1186/s12885-015-1959-5.

(2) Castle, P. E.; Eaton, B.; Reid, J.; Getman, D.; Dockter, J. Comparison of Human Papillomavirus Detection by Aptima HPV and Cobas HPV Tests in a Population of Women Referred for Colposcopy Following Detection of Atypical Squamous Cells of Undetermined Significance by Pap Cytology. *J Clin Microbiol* **2015**, *53* (4), 1277–1281. https://doi.org/10.1128/JCM.03558-14.

(3) Oštrbenk Valenčak, A.; Kroon, K. R.; Fabjan, D.; Mlakar, J.; Seme, K.; Berkhof, J.; Poljak, M. Clinically Validated HPV Assays Offer Comparable Long-term Safety in Primary Cervical Cancer Screening: A 9-year Follow-up of a Population-based Screening Cohort. *Intl Journal of Cancer* **2025**, *156* (4), 788–801. https://doi.org/10.1002/ijc.35200.

(4) Toliman, P. J.; Kaldor, J. M.; Badman, S. G.; Phillips, S.; Tan, G.; Brotherton, J. M. L.; Saville, M.; Vallely, A. J.; Tabrizi, S. N. Evaluation of Self-Collected Vaginal Specimens for the Detection of High-Risk Human Papillomavirus Infection and the Prediction of High-Grade Cervical Intraepithelial Lesions in a High-Burden, Low-Resource Setting. *Clinical Microbiology and Infection* **2019**, *25* (4), 496–503. https://doi.org/10.1016/j.cmi.2018.05.025.

(5) Kundrod, K. A.; Smith, C. A.; Hunt, B.; Schwarz, R. A.; Schmeler, K.; Richards-Kortum, R. Advances in Technologies for Cervical Cancer Detection in Low-Resource Settings. *Expert Review of Molecular Diagnostics* **2019**, *19* (8), 695–714. https://doi.org/10.1080/14737159.2019.1648213.

(6) Bordigoni, A.; Motte, A.; Tissot-Dupont, H.; Colson, P.; Desnues, C. Development and Validation of a Multiplex qPCR Assay for Detection and Relative Quantification of HPV16 and HPV18 E6 and E7 Oncogenes. *Sci Rep* **2021**, *11* (1), 4039. https://doi.org/10.1038/s41598-021-83489-2.
